# Dissecting the dynamics of signaling events in the BMP, WNT, and NODAL cascade during self-organized fate patterning in human gastruloids

**DOI:** 10.1371/journal.pbio.3000498

**Published:** 2019-10-15

**Authors:** Sapna Chhabra, Lizhong Liu, Ryan Goh, Xiangyu Kong, Aryeh Warmflash

**Affiliations:** 1 Systems, Synthetic and Physical Biology, Rice University, Houston, Texas, United States of America; 2 Department of Biosciences, Rice University, Houston, Texas, United States of America; 3 Department of Mathematics, Boston University, Boston, Massachusetts, United States of America; 4 Department of Bioengineering, Rice University, Houston, Texas, United States of America; University of Edinburgh, UNITED KINGDOM

## Abstract

During gastrulation, the pluripotent epiblast self-organizes into the 3 germ layers—endoderm, mesoderm and ectoderm, which eventually form the entire embryo. Decades of research in the mouse embryo have revealed that a signaling cascade involving the Bone Morphogenic Protein (BMP), WNT, and NODAL pathways is necessary for gastrulation. In vivo, WNT and NODAL ligands are expressed near the site of gastrulation in the posterior of the embryo, and knockout of these ligands leads to a failure to gastrulate. These data have led to the prevailing view that a signaling gradient in WNT and NODAL underlies patterning during gastrulation; however, the activities of these pathways in space and time have never been directly observed. In this study, we quantify BMP, WNT, and NODAL signaling dynamics in an in vitro model of human gastrulation. Our data suggest that BMP signaling initiates waves of WNT and NODAL signaling activity that move toward the colony center at a constant rate. Using a simple mathematical model, we show that this wave-like behavior is inconsistent with a reaction-diffusion–based Turing system, indicating that there is no stable signaling gradient of WNT/NODAL. Instead, the final signaling state is homogeneous, and spatial differences arise only from boundary effects. We further show that the durations of WNT and NODAL signaling control mesoderm differentiation, while the duration of BMP signaling controls differentiation of CDX2-positive extra-embryonic cells. The identity of these extra-embryonic cells has been controversial, and we use RNA sequencing (RNA-seq) to obtain their transcriptomes and show that they closely resemble human trophoblast cells in vivo. The domain of BMP signaling is identical to the domain of differentiation of these trophoblast-like cells; however, neither WNT nor NODAL forms a spatial pattern that maps directly to the mesodermal region, suggesting that mesoderm differentiation is controlled dynamically by the combinatorial effect of multiple signals. We synthesize our data into a mathematical model that accurately recapitulates signaling dynamics and predicts cell fate patterning upon chemical and physical perturbations. Taken together, our study shows that the dynamics of signaling events in the BMP, WNT, and NODAL cascade in the absence of a stable signaling gradient control fate patterning of human gastruloids.

## Abstract 2

A self-organizing in vitro model of cell fate decisions during human gastrulation reveals that patterning is controlled by combinatorial dynamics of multiple signaling pathways, rather than by stable gradients.

## Introduction

Gastrulation is a crucial stage in embryonic development when a homogeneous population of pluripotent epiblast cells self-organizes to form the 3 germ layers: endoderm, mesoderm, and ectoderm, which develop into the embryo. Insights into mammalian gastrulation come from decades of genetic and biochemical studies in the mouse embryo [1]. These studies have revealed that a signaling cascade involving the Bone Morphogenic Protein (BMP), WNT, and NODAL pathways is integral for initiating gastrulation. BMP signaling in the extra-embryonic ectoderm activates WNT signaling in the epiblast and the overlying visceral endoderm [2,3]. WNT signaling activates NODAL signaling in these two tissues, and NODAL signaling in turn feeds back to maintain BMP signaling in the extra-embryonic ectoderm [3,4]. This circular signaling cascade from BMP to WNT to NODAL, and back to BMP, initiates the formation of primitive streak—the site of germ layer formation, at the posterior end of the embryo.

This picture of gastrulation in which these pathways induce the primitive streak is based on studies that show that ligands for these pathways are expressed on the posterior side of the embryo and that removal of the ligands or other pathway components leads to loss of the primitive streak or some of its derivatives [5–8]. Measurements of signaling activity dynamics, as reflected in signal transducers—nuclear β-catenin for WNT and nuclear SMAD proteins for BMP or NODAL—have not been performed in gastrulation-staged embryos. Available data are thus consistent with the simplest model in which relatively stable signaling gradients are formed and then interpreted by cells to decide cell fates. However, other models are also possible. Signaling patterns may be dynamic, and cells may use various strategies to interpret these dynamics [9–12]. Differentiating between these models requires determining the spatiotemporal signaling dynamics, their regulation, and the mechanisms that translate signaling activities to cell fate patterning during gastrulation.

Theoretical studies provide plausible mechanisms for self-organized fate patterning. In 1952, Alan Turing proposed a model for how diffusible signals could lead to self-organized patterning during development. In this model, a steady state with homogenous expression becomes unstable when the extracellular signals are allowed to diffuse, and the system evolves to a new steady state with a spatial pattern. This process is known as a diffusion-driven, or Turing, instability [13]. Since then, a particular molecular realization of such an instability, in which a diffusible activator activates both itself and its inhibitor, has been proposed to explain a diverse range of biological processes [14–17]. Experimentally, reaction-diffusion–based Turing systems have been shown to underlie hair follicle spacing, digit patterning, and generation of left-right asymmetry during mouse development [18–20]. Interestingly, WNT forms a part of the Turing system active in hair follicle spacing and digit patterning, and NODAL forms a Turing system in left-right asymmetry. Due to the technical challenges of studying gastrulation in vivo, whether WNT or NODAL generates a Turing instability to establish a signaling gradient during gastrulation remains unknown.

In a previous study, we showed that spatially confined human embryonic stem cells (hESCs) treated with BMP4 self-organize to form radial patterns of distinct germ layers: an outer ring of extra-embryonic cells, followed by endodermal and mesodermal rings, and an ectodermal center, thus recapitulating some aspects of gastrulation in vitro [21] (hereafter referred to as gastruloids). These findings have since been reproduced in other labs [22,23], and a comparable system for mouse embryonic stem cells has been developed [24]. Three-dimensional models have also been developed that recapitulate aspects of early mammalian development [25–28] and, in some cases, even morphologically resemble embryos [29–31]. Although the germ layer patterns in gastruloids differ from the trilaminar germ layer patterns in vivo, they offer a reproducible, quantitative, and controlled system to examine mechanisms underlying early embryonic patterning.

Recent studies have provided insights into the mechanisms underlying the self-organized fate patterning of gastruloids. Exogenous BMP4 initially activates signaling homogeneously throughout the colony, but within 12 h, signaling is restricted to the colony edges [10]. This restriction is caused by up-regulation of the BMP inhibitor NOGGIN together with a lack of BMP receptor accessibility at the colony center [32]. In response to BMP treatment, hESCs activate endogenous WNT and NODAL signaling in a sequential order (BMP→WNT→NODAL) as observed during mouse gastrulation [33]. In contrast with BMP signaling, NODAL signaling activity is highly dynamic: a wave of signaling moves from the edge toward the colony center at a constant rate, specifying the mesendodermal rings in its wake [10]. While BMP signaling at the edge controls extra-embryonic differentiation, WNT and NODAL control mesendodermal differentiation [21,33]. However, unlike BMP signaling, which can be spatially mapped to extra-embryonic differentiation, NODAL signaling activity extends farther than the domain of mesendodermal differentiation [10], suggesting that a threshold level of NODAL signaling alone is insufficient to determine the position of mesendodermal differentiation. The mechanisms governing the spatial extent of mesendodermal differentiation and the wave-like activity of NODAL signaling remain largely unknown.

In this study, we reevaluated the requirements for the BMP, WNT, and NODAL pathways in fate patterning; examined the mechanisms underlying their signaling dynamics; and evaluated signaling features that correlate with fate patterning. Our results demonstrate that BMP signaling triggers waves of WNT and NODAL signaling activities, which move toward the colony center at a constant rate. Mathematical modeling revealed that these waves are inconsistent with a reaction-diffusion based Turing system, suggesting that neither WNT nor NODAL forms a stable spatial gradient of signaling activity. We further show that although longer durations of WNT and NODAL signaling promote mesoderm differentiation, neither of them forms a signaling pattern that spatially maps to the mesodermal region, suggesting that mesoderm differentiation is controlled dynamically by combinatorial signaling through multiple pathways. Finally, we synthesize these data into a mathematical model that predicts signaling dynamics and subsequent fate patterning under chemical and geometric perturbations. Taken together, our data suggest that the dynamics of signaling events in the cascade involving BMP, WNT, and NODAL pathways—and not a signaling gradient—controls the self-organized fate patterning of human gastruloids.

## Results

### Gastruloids spatially pattern into extraembryonic, mesendodermal, and pluripotent cells in defined media

**Fig 1:**
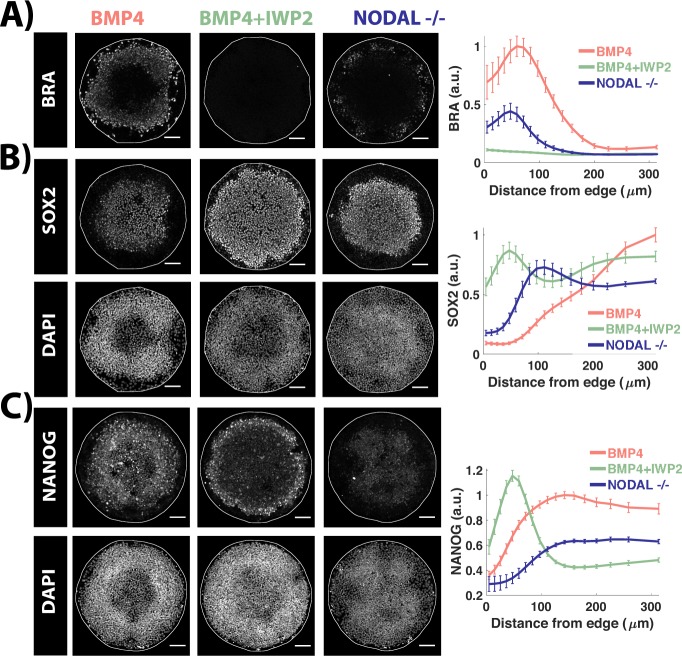
WNT initiates and NODAL up-regulates mesodermal differentiation. (A–C) Images of samples immunostained for the indicated markers at 44 h post BMP treatment in different conditions: control and *NODAL* −/− cells were treated with 50 ng/ml BMP4. BMP+IWP2 represents wild-type cells treated with 50 ng/ml BMP and 5 μM IWP2. Quantification represents intensity levels of indicated markers normalized to DAPI, averaged at different positions along the colony radii. Error bars represent standard error of the mean. *N* ≥ 10. Scale bars = 100 μm. Colony diameter = 700 μm. Underlying data can be found in S1 Data. Data structure explanation can be found in S1 Text. BMP, Bone Morphogenic Protein.

To examine the role of paracrine signals without influence from undefined components present in mouse embryonic fibroblast conditioned media (MEF-CM), we performed the micropatterned gastrulation assay in the defined mTeSR1 media as outlined by Deglincerti and colleagues[34]. In mTeSR1, hESCs treated with BMP4 self-organize to form an outer ring of CDX2+ extra-embryonic cells and an inner ring of BRACHYURY (BRA+) primitive streak or mesodermal cells (Fig 1A). SOX17+ endodermal cells fall in between these two (S1A Fig), as observed previously in MEF-CM [21]. Inside of the BRA+ ring, a disc of cells at the colony center co-expresses the pluripotency markers NANOG and SOX2 (Fig 1B and 1C). Compared to the MEF-CM protocol in which NANOG expression was lost in the center cells [21], the mTeSR1 protocol recapitulates an earlier time point in gastrulation when primitive streak formation has begun, but the remainder of the epiblast remains pluripotent with only shallow gradients in pluripotency markers such as NANOG [1,24].

### WNT signaling initiates and NODAL signaling up-regulates mesodermal differentiation in the ring-like domain

To determine the role of paracrine WNT and NODAL signaling in this self-organized differentiation, we first inhibited these signals by chemical or genetic perturbations. To inhibit WNT signaling, we added IWP2, which inhibits the secretion of all WNT ligands [35], at the beginning of the gastrulation assay. To inhibit NODAL signaling, we created NODAL knockout cells (*NODAL*−/−) using CRISPR-Cas9 and used these cells in the gastrulation assay. Despite the absence of a functional NODAL protein (S2A and S2C Fig), these cells remain pluripotent for over 15 passages (S2B Fig) due to the presence of exogenous Transforming Growth Factor β1 (TGFβ1) in mTeSR1 media. Thus, although cells remain competent to respond, cell-to-cell signaling by the NODAL ligand is lost.

Studies in various model organisms and in vitro systems have established the necessity of WNT and NODAL signaling for the formation of primitive streak, where mesoderm and endoderm cells originate [1,36]. Consistent with this, removing either WNT or NODAL signaling reduces mesoderm differentiation, quantified as the average BRA intensity as a function of distance from the colony edge (Fig 1A). Because our current imaging resolution does not permit segmentation of single cells, we cannot determine quantitatively whether the reduction entails reduced number of BRA+ cells or reduced BRA intensity across all cells. Qualitatively, the reduction involves both effects.

Unlike NODAL inhibition, WNT inhibition completely abolishes mesodermal differentiation (Fig 1A). Inhibiting the NODAL pathway activity downstream of ligand-receptor binding using a small molecule inhibitor of receptor kinase activity, SB431542 (SB) [37], has a more severe effect on mesodermal differentiation, as shown previously [21,33] (S1D and S1E Fig). Consistent with this, NODAL knockout cells show high nuclear SMAD2—a signal transducer of active NODAL/TGF-β signaling—at the colony edges, which is lost upon treatment with SB (S1C Fig). However, even inhibition of both endogenous and exogenous NODAL signals with SB does not result in as complete an inhibition of differentiation as that observed upon inhibition of WNT signaling. This suggests that WNT signaling initiates mesoderm differentiation, whereas NODAL signaling up-regulates it. Endoderm differentiation, on the other hand, requires both WNT and NODAL signaling for its initiation as inhibition of either pathway completely abolished SOX17 expression (S1A and S1D Fig).

### WNT and NODAL signaling maintain high NANOG levels at the colony center

Surprisingly, WNT inhibition reduces NANOG protein levels at the colony center while maintaining NANOG levels in a ring-like domain closer to colony edge, where cells co-express NANOG with SOX2 (Fig 1C). Because *NANOG* is a transcriptional target of NODAL signaling [38], its restriction near the colony edges could be a result of NODAL signaling being high there. In contrast to this, NANOG protein expression in the *NODAL*−/− cells treated with BMP4 extends all the way to the colony center, although the expression levels are low compared to the NANOG expression in wild-type cells treated with BMP4 (Fig 1C), suggesting that NODAL is necessary to achieve high levels of NANOG protein expression in the colony center. In addition to NODAL secreted by cells, TGF-β present in mTeSR1 also activates NODAL signaling, and inhibition of NODAL pathway activity downstream of ligand-receptor binding using SB severely reduces NANOG expression in the colonies (S1E Fig), consistent with previous studies that show that NODAL signaling is necessary for maintaining pluripotency of hESCs [39,40]. Taken together, these results indicate that WNT and NODAL signaling synergize to maintain NANOG expression in the colony center while the outer layers of the colony are patterned into mesendoderm and extraembryonic cells.

### BMP signaling drives extra-embryonic differentiation at the colony edge independent of WNT and NODAL signaling

**Fig 2:**
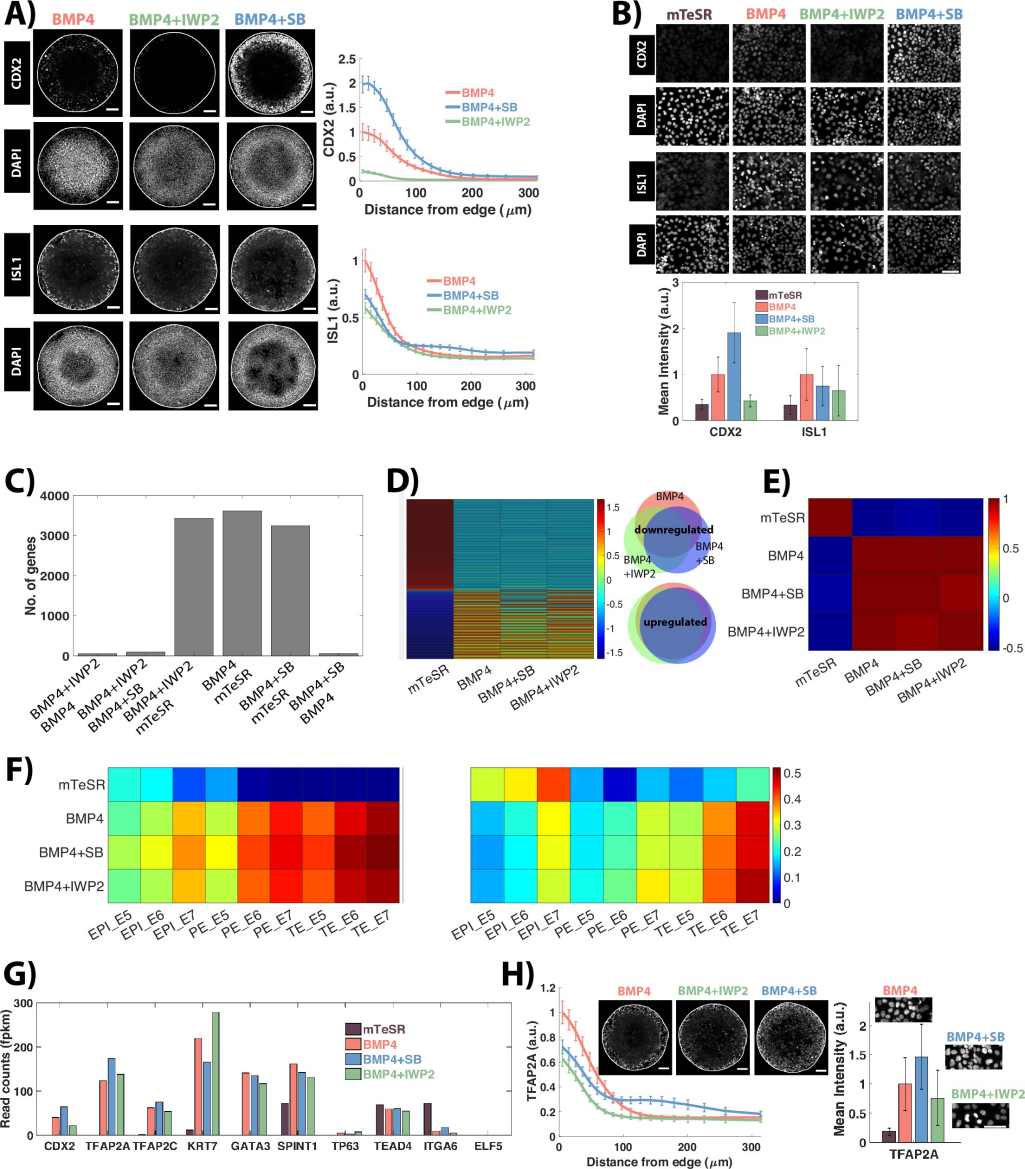
BMP controls the differentiation of CDX2+ edge cells, which transcriptionally resemble in vivo human trophoblast cells. (A) Images of samples immunostained for the indicated markers at 44 h post BMP treatment in different conditions—BMP4 alone, BMP and IWP2, and BMP4 and SB. Quantification represents intensity levels of indicated markers normalized to DAPI, averaged at different positions along the colony radii. Error bars represent standard error of the mean. *N* ≥ 10. (B) Images of cells grown in standard conditions in sparse culture and immunostained for the indicated markers at 48 h post BMP4 treatment in different conditions. No BMP4 was added to the mTeSR sample. Quantification represents average mean intensity levels per cell of indicated markers normalized to DAPI. Cells *N* > 500. Error bars represent standard deviation across cells. (C) Number of differentially expressed genes (*p* ≤ 0.05, fold change ≥ 2, FPKM ≥ 1 in at least one sample of the 4 samples) between indicated samples. (D) (Left) Heatmap showing z-scores of 284 most differentially expressed genes as defined in the text. (Right) Venn diagram of differentially down-regulated/up-regulated genes between indicated samples as compared to mTeSR1. Red: BMP4, Green: BMP4 + IWP2, Blue: BMP4 + SB. (E) Pearson correlation coefficients for normalized read count values (FPKM) of 284 differentially expressed genes between indicated samples. (F) (Left) Pearson correlation coefficients between indicated samples for z-scores of 284 differentially expressed genes determined from hESC samples. (Right). Pearson correlation coefficients between indicated samples for z-scores of 174 lineage-specific genes determined from in vivo human embryo data. (G) Normalized read count values, expressed in FPKM, of common trophoblast markers in the indicated samples. (H) Images of samples immunostained for TFAP2A in micropatterns, and regular culture. Quantification as defined in (A). *N* ≥ 10 colonies for micropatterns, *N* > 500 cells for regular culture. Scale bar = 100 μm. Underlying data can be found in S2 Data. BMP, Bone Morphogenic Protein; E5/E6/E7, embryonic day 5/6/7 (respectively); EPI, epiblast; FPKM, fragments per kilobase of transcript per million mapped reads; hESC, human embryonic stem cell; PE, primitive endoderm; SB, SB431542; TE, trophectoderm.

Loss of paracrine WNT signaling results in the loss of CDX2 protein expression in cells at the colony edges (Fig 2A). This is surprising because WNT signaling is dispensable for trophectoderm development in the mouse embryo [41] and WNT inhibition promotes trophectoderm differentiation from hESCs in vitro [42]. These cells are also negative for SOX2, NANOG, and BRA (Fig 1), suggesting that they have left the pluripotent state but are not differentiating to mesendoderm. To further determine the identity of these cells, we examined the transcriptomes of sparsely seeded hESCs differentiated in standard culture, which adopt the same fate as cells on the edges of gastruloids under several different differentiation conditions (Figs 2B and S3A). We performed bulk RNA sequencing (RNA-seq) on hESCs under 4 conditions: untreated pluripotent cells in mTeSR1 and cells treated with either BMP4, BMP4 + SB, or BMP4 + IWP2 for 48 h, and compared their transcriptomes to each other and to published human embryo RNA-seq datasets.

Differential gene expression analyses within the samples revealed that over 3,000 genes differ at least 2-fold in expression in each of the 3 BMP-treated conditions (BMP alone, BMP + SB, and BMP + IWP2) compared with the pluripotent condition. The number of differentially expressed genes between either of the 3 treatment conditions is much lower (<100), and the fold changes are smaller (Figs 2C and S3C). This suggests that the 3 treatment conditions are more different from pluripotent cells than from each other. To further examine the similarity between the 3 treatment conditions, we analyzed genes that are differentially expressed in either of these conditions compared to the pluripotent cells. We formulated a subset of these genes that comprise the union of the first 100 (ranked according to fold change) differentially up-regulated and the first 100 differentially down-regulated genes in each of the 3 conditions. This gave a total of 284 differentially expressed genes (S1 Table). Intriguingly, there is a large overlap in this subset of differentially expressed genes: 77 genes are up-regulated, and 54 genes are down-regulated in all 3 conditions (Fig 2D). The expression values of the differentially expressed genes are also highly correlated (Pearson correlation coefficient [r] > 0.9) (Fig 2E). Taken together, these results suggest that despite different CDX2 protein levels, the edge populations under the 3 treatment conditions possess a highly similar transcriptome and therefore likely represent similar differentiated states. Future studies will be necessary to determine whether these differences in CDX2 levels are indicative of distinct developmental time points or differentiated subtypes or, alternatively, whether they do not correspond to different cell states.

### Extra-embryonic cells at the colony edge are transcriptionally similar to trophoblast cells in the human embryo

The in vivo cellular identity of BMP-treated hESCs has remained controversial, with some studies suggesting a trophoblast cell state [43] and others suggesting an extra-embryonic mesodermal state [44]. Here, we compare the transcriptome of these cells with previously published pre-implantation human embryo datasets to determine their similarity with human trophectoderm.

We first compared the expression patterns of 284 genes differentially expressed in all 3 BMP-treated samples in our dataset with expression patterns in the different stages of pre-implantation human embryo published in a previous study [45]. All samples show the highest correlation with trophectodermal cells, indicating a similar expression profile of BMP signaling targets and pluripotency genes (Fig 2F). Next, we identified a set of lineage-specific genes that define the 3 lineages (epiblast, primitive endoderm [PE], and trophectoderm) of the blastocyst-staged human embryo (S3D Fig) and compared the expression of these genes in human embryos with that in our dataset. To determine lineage-specific genes, we first extracted genes that are differentially expressed between that lineage and at least one of the other 2 lineages (fold change > 5, false discovery rate [FDR] = 0.01). From this list, we excluded genes that are differentially expressed between different time points within that lineage (embryonic day [E]5, E6, E7) to reduce noise within the lineage and further removed genes with a low expression value (Reads Per Kilobase of transcript per Million mapped reads [RPKM] < 10 in at least 2 of the 3 time points for that lineage). This gave a list of 174 lineage-specific genes (S1 Table). We then evaluated the correlations in the expression of these genes between the embryo data and the hESC samples. As before, all 3 BMP-treated samples show highest correlation with trophectodermal cells, indicating a similar expression profile (Fig 2G).

Along with an up-regulation of pre-implantation trophectoderm markers—*CDX2*, *GATA3*, *TFAP2C*, and *KRT7* [46]—BMP-treated cells also display an elevated expression level of *TFAP2A*, *TP63*, and *SPINT1*, genes that are commonly expressed in the week 6 cytotrophoblast (CT) cells of the human placenta [47,48] (Fig 2H). Using immunostaining, we further verified the expression of both GATA3 and TFAP2A at the protein level (Figs 2I, S3A and S3B). Other well-established CT markers, such as *ELF5*, are not expressed in BMP-treated cells [48]. It is important to note that *ELF5* is also not expressed in the preimplantation human trophoblast [46]. Taken together, these data suggest that all 3 BMP-treated conditions represent a trophoblast cell type, most likely between weeks 2 and 6 of human development.

In primates, including monkey and humans, extra-embryonic mesoderm is present prior to the formation of the primitive streak [49,50] and thus cannot be solely derived from the primitive streak, as in the mouse [51]. In single-cell RNA-seq data from monkey embryos at primitive streak stages, only this nonprimitive streak-derived population is present. These cells transcriptionally resemble the PE and express well-established PE markers—*GATA4* and *GATA6*—indicating a PE origin [52]. BMP-treated hESCs, in contrast, do not express any of these markers (S3E Fig) and are transcriptionally more similar to trophectoderm cells than PE cells (Fig 2F and 2G), thus arguing against their identification as extra-embryonic mesoderm.

### WNT and NODAL signaling dynamics lie outside the Turing instability regime

Having established the necessity of WNT and NODAL signaling for the fate patterning of human gastruloids, we next examined the dynamics of signaling and the mechanisms of signal propagation through these 2 pathways. Both WNT and NODAL signaling are known to activate the production of their own ligands and inhibitors [7,53], and previous results show that these inhibitors are essential for fate patterning [21, 28, 32]. Theoretical work has shown that activator-inhibitor motifs acting in reaction-diffusion systems, can, under certain conditions, enable pattern formation in a homogeneous system. The patterns arise due to a diffusion-driven instability (also called Turing instability) and exhibit a fixed wavelength [13–15, 17, 54].

**Fig 3:**
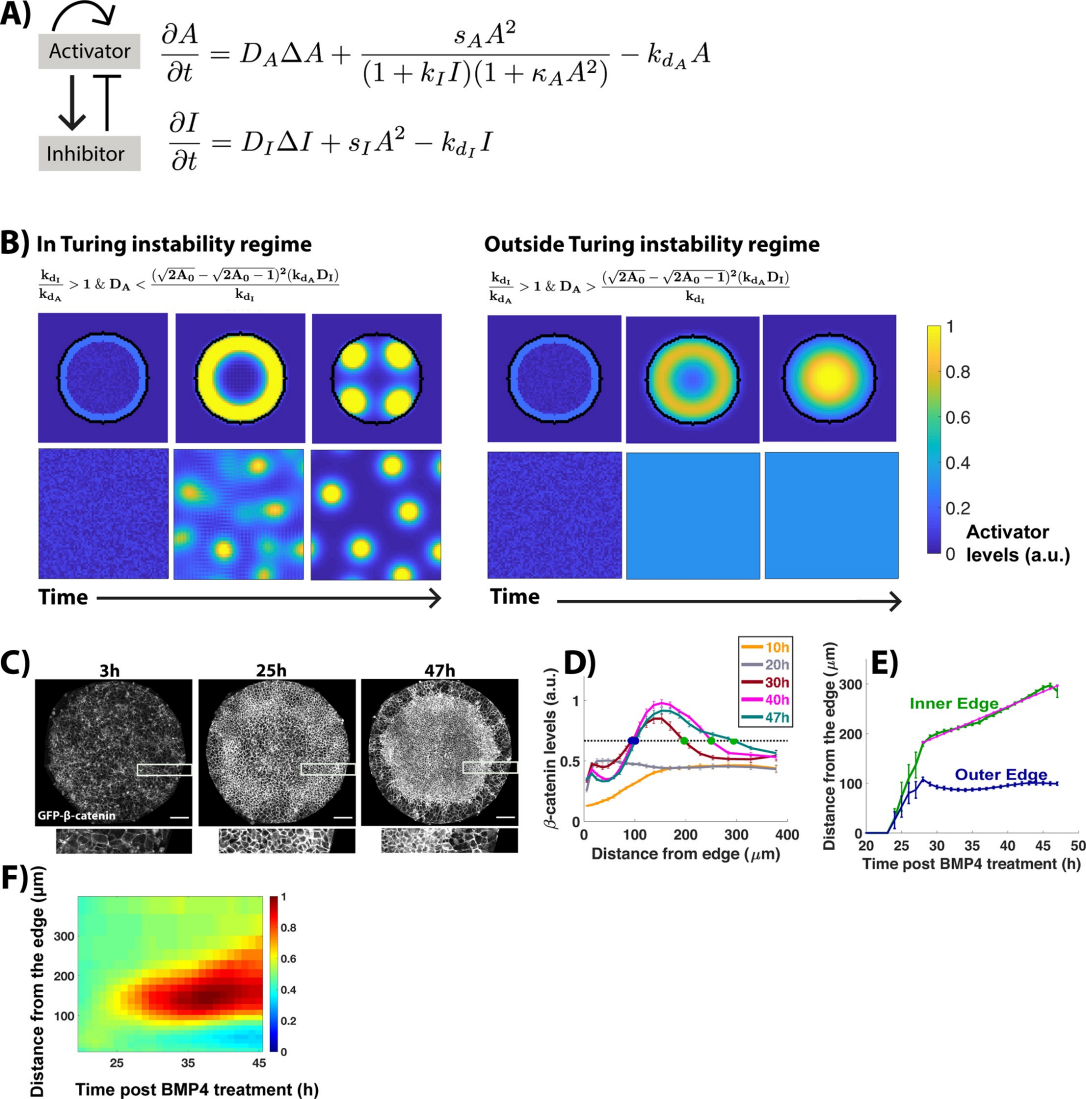
WNT signaling dynamics lie outside the Turing instability regime. (A) Schematic and equations of the model. (B) Evolution of activator levels from the initial state to the steady state. Inequalities define parameter regimes that display each of the 2 behaviors (S1 Model). In each case, the top row shows the model simulated on a circular colony, while the bottom shows it simulated in a domain with cells throughout. Simulation parameters: s_A_ = 0.01, k_I_ = 1, k_A_ = 0, kd_A_ = 0.001, D_I_ = 0.4, s_I_ = 0.01, kd_I_ = 0.008. (left) D_A_ = 0.014, (right) D_A_ = 0.0025 for outside and inside Turing instability regime, respectively. Simulation domain: 190 × 190 pixel square lattice with periodic boundary conditions and random distribution of activator/inhibitor as initial conditions. To simulate the model in circular colonies, a circle (radius: 25 pixels) is defined at the center of the lattice. Outside the circle, s_A_ = s_I_ = 0, and kd = 0.01. (C) Snapshots of GFP-β-catenin hESCs from time-lapse imaging at indicated time points post BMP treatment. Marked regions are magnified in images shown below. (D) Average nonmembrane β-catenin intensity levels as a function of distance from the colony edge. The legend indicates the time post BMP treatment represented by each curve. Green and blue dots represent the front and back edges of the active signaling domain, respectively. (E) The positions of the inner and outer edge of the signaling domain as a function of time post BMP addition. Magenta line shows linear fit with equation 6.02 μm/h × t + 13.57 μm, R^2^ = 0.98. (F) Kymograph showing spatiotemporal evolution of nonmembrane β-catenin levels. At time points earlier than the first time plotted in D, signaling is below threshold signaling at all positions in the colony. Scale bar = 100 μm. Underlying data can be found in S3 Data. BMP; Bone Morphogenic Protein; GFP; Green Fluorescent Protein; hESC, human embryonic stem cell.

To determine whether either WNT or NODAL signaling compose a reaction-diffusion system with a Turing instability, we first used a mathematical model to predict the dynamics of an activator-inhibitor system on a confined colony with and without a Turing instability, and then compared it with the experimentally measured signaling dynamics for WNT and NODAL. This model is composed of a simple activator-inhibitor motif and is not intended to specifically simulate WNT or NODAL. Within this activator-inhibitor model, we analytically derived the conditions for a pattern to develop due to a Turing instability. When these conditions fail to be satisfied, the system remains in a stable homogeneous state (S1 Model). We then simulated this activator-inhibitor model in both regimes. To avoid the effects of boundary conditions, we simulated the colony in a larger lattice with protein production only within the colony but diffusion and protein degradation throughout the lattice. We started with initial conditions of high activator levels at the colony border, which reflects the position of NODAL signaling initiation [10]. The simulations show that within the Turing instability regime, the activator that starts at the boundary eventually creates either stable circular spots or a stripe of fixed width at the boundary, with the shape depending on the parameters used (Figs 3A, 3B and S4A, S1–S6 Movies). On the other hand, outside the Turing instability regime, the activator expands toward the colony center (Fig 3B; S3 and S4 Movies). This expansion occurs due to autoactivation and diffusion of the activator, and the differences between the edge and center of the colony at steady state result from diffusive loss of signals at the colony boundary. Thus, within the Turing instability regime, the activator evolves to a steady-state territory of a fixed size near its original position of activation.

Our previous data on NODAL signaling dynamics show that NODAL signaling starts at the colony edge and continuously expands inward at a constant rate [10]. In some colonies, NODAL signaling reaches all the way to the colony center and reduces at the colony edges where it initially started (S1C Fig). This behavior is consistent with the behavior of activator-inhibitor models outside the Turing regime. It would only be consistent with the behavior inside the Turing regime if the length scale of the patterns created were larger than the colony. In this case, although the system would technically contain a Turing instability, it would not be relevant to the fate patterns formed.

We next examined the dynamics of WNT signaling using time-lapse microscopy of transgenic hESCs with Green Fluorescent Protein (GFP) inserted into the endogenous β-catenin locus (GFP-β-catenin hESCs [55]). These cells were seeded onto micropatterned colonies and imaged from 3 to 47 h post BMP4 treatment. β-catenin is stabilized by WNT signaling and serves as a transcription factor to activate WNT pathway targets. However, β-catenin also localizes to adherens junctions at the cell membrane. To avoid misinterpreting signaling activity due to the membrane population of β-catenin, we considered only nonmembrane fluorescence as a measure of WNT signaling activity. In the first 20 h following treatment, the colonies initially contract in response to withdrawal of Rock-Inhibitor and then spread to fill the entire micropatterned space available. During this time, WNT signaling increases throughout the colony, with the colony edges showing slightly higher signaling than the rest. From 20 to 40 h, the peak signaling increases continuously, but from 40 to 47 h, it decreases to a slightly lower value. We quantified the spatial dynamics of WNT signaling by defining a territory of active signaling as the region with nonmembrane β-catenin levels greater than half the maximal WNT signaling in the entire time course and traced the position of this territory in time (S4C Fig). We note that this definition is a convenient way to quantify the spatial extent of WNT signaling, but none of our conclusions depend on the choice of threshold for active signaling. Active signaling first occurs near colony edges between 24 h and 27 h. From 27 h onward, the active signaling forms a ring-like domain the inner edge of which moves toward the colony center at a constant rate, while the outer edge remains stationary, resulting in continuous expansion of the area of active WNT signaling (Figs 3C–3F and S4B, S7 Movie).

As with NODAL, WNT dynamics are inconsistent with a Turing instability. It is important to note that although we model a simple activator-inhibitor motif, without specific pathway interactions, the results apply to more complex models as well. This is because a Turing instability will always result in patterns with a fixed wavelength. Under certain conditions, it is possible to achieve a wave-like behavior with a Turing instability, but the wave is oscillatory in nature [56]. A wave that expands inward, as observed for WNT and NODAL, either lies outside the Turing instability regime or represents a Turing instability with a wavelength greater than the size of the colony. In both cases, the Turing instability is irrelevant for the fate patterns observed.

To sum, a Turing instability does not explain the time evolution of WNT and NODAL signaling. The expanding wave-like behavior of signaling is consistent with initial activation at the colony boundary, and the system moving toward a homogenous state of WNT and NODAL activity throughout the colony. Steady-state signaling differences between the edge and the rest of colony arise only due to boundary effects.

### Inward movement of WNT and NODAL signaling activities is not due to cell movement

**Fig 4:**
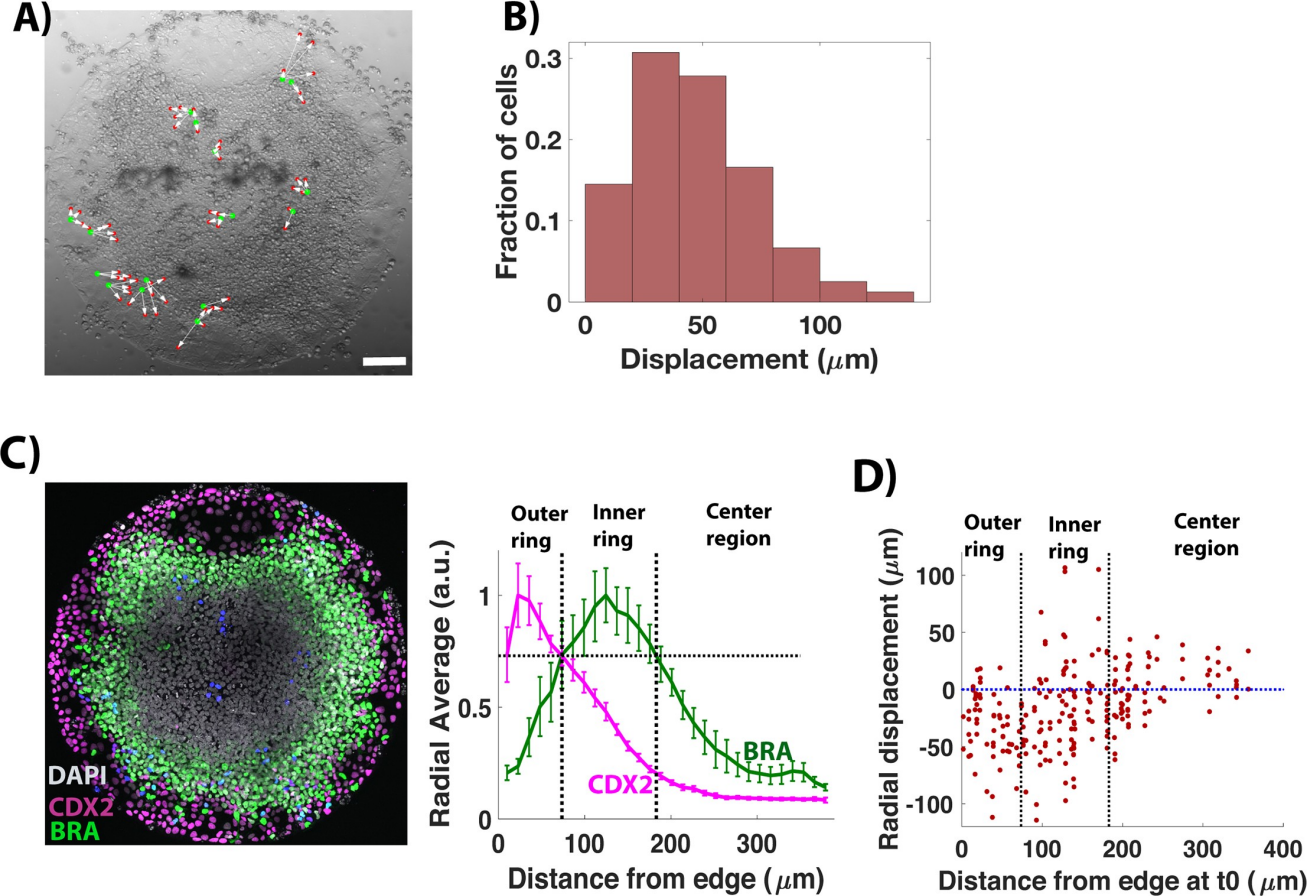
Inward movement of signaling activities is not due to cell movement. (A) Snapshot from time-lapse imaging at 47 h post BMP treatment in the bright field channel. Green and red dots represent initial and final positions, respectively, of labeled cells that were correctly tracked throughout imaging. (B) Histogram of displacement of tracked cells. *n* = 84 cells. (C) Image of colony (shown in A) immunostained for CDX2, BRA post live cell imaging. Quantification represents average nuclear intensities of indicated markers normalized to DAPI as a function of radial position. Error bars represent standard error of the mean. *N* = 6. The lines indicate the inner and outer positions of a region of high BRA as a rough guide to the different fate territories (D) Radial displacement of cells as a function of their starting position. Radial displacement = distance of the cell from the center at end of imaging − distance of the cell from the center at the start of imaging. The vertical dotted lines are in the same positions as in (C). The horizontal dashed line separates the cells moving toward the edge from cells moving toward the center. Underlying data can be found in S4 Data. BMP, Bone Morphogenic Protein; BRA, BRACHYURY.

Next, we examined the extent to which active cell migration contributes to the inward movement of signaling activities by tracking the movement of individual cells during fate patterning. To improve tracking efficiency, we mixed cells labeled with a VENUS-H2B fusion protein with unlabeled cells. We verified that there is no phase separation between the two cell types by imaging well-mixed populations of these cells and comparing them with control cell populations that sort into separate domains (S5A Fig). Then, we performed the gastrulation assay with a mixed population of VENUS-H2B cells and unlabeled cells (labeled:unlabeled = 1:100), and imaged them during the inward spreading of WNT signaling activity (20–47.5 h post BMP treatment (Fig 4A and 4C, S8 Movie).

Quantifying cell movement revealed that the displacement of cells averages only about two cell diameters from the starting position (Fig 4B). Although cells in the outer region of the colony move inward (Fig 4D), the physical movement of cells occurs over a smaller distance than the movement of signaling domains, with both WNT and NODAL signaling domain moving at least 100 μm inward during this time (Fig 3E and 3F; [10]), indicating that active cell migration plays a limited role in signaling movement. Quantifying the progeny of tracked cells revealed that the cell division rates across the colony (S5B and S5C Fig) are similar, indicating an absence of differential cell growth during fate patterning.

### BMP activates WNT, which activates NODAL signaling during fate patterning

Prior studies have shown that a signaling cascade in which BMP activates WNT, which subsequently activates NODAL (BMP→WNT→NODAL), is integral to initiating gastrulation in the mouse embryo and is also active during in vitro human gastrulation in a different protocol [1,33]. We next examined the role of this signaling cascade in the formation of WNT and NODAL waves and further evaluated whether there is feedback of WNT and NODAL on upstream signals in the cascade.

**Fig 5:**
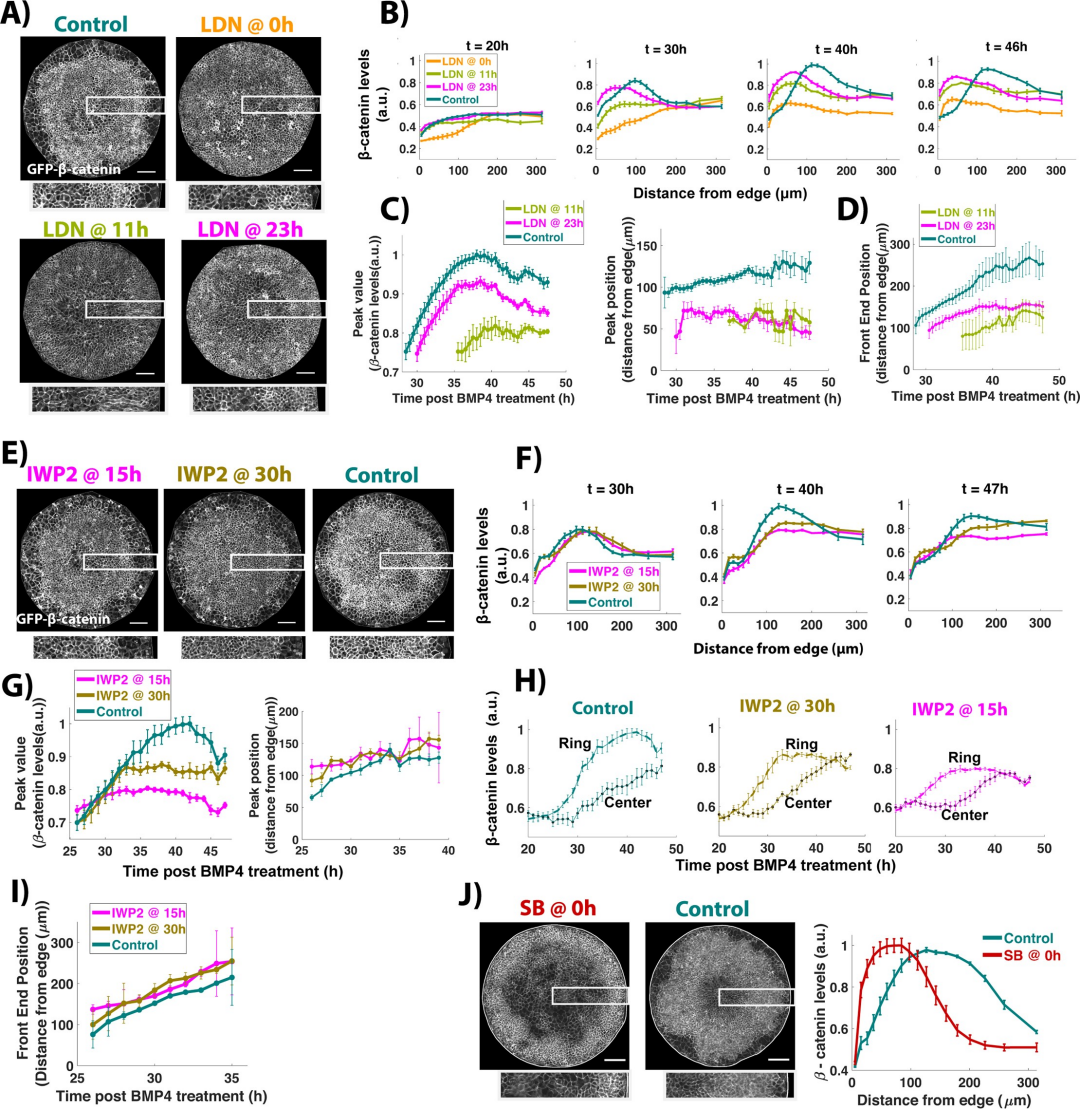
Regulation of WNT signaling activity by BMP, WNT, and NODAL. (A, E) Snapshots of GFP-β-catenin hESCs from time-lapse imaging movies at 46 h post BMP treatment. Time between BMP4 and LDN or IWP2 addition is indicated above each image. Boxes show the region represented at higher magnification below each colony image. (B, F) Average nonmembrane β-catenin levels as a function of radial position. Control represents samples with no inhibitor addition. The timing of LDN or IWP2 addition after BMP4 treatment for each curve is shown in the legend, while the time being analyzed in shown above the plot. Error bars represent standard error. *N* = 3 colonies for the samples LDN at 0 h (LDN@0h), LDN at 11 h (LDN@11h); for all other samples *N* ≥ 5. (C, G) Temporal evolution of the position and intensity of peak signaling (defined by maximal nonmembrane β-catenin intensity). (D, I) Temporal evolution of the front of the domain of active signaling. In (C, D, G, H, and I) at time points earlier than the first one in each curve, signaling was below threshold signaling at all positions. (H) Temporal evolution of average nonmembrane β-catenin levels in a narrow ring inside the region of mesodermal differentiation (distance from edge: 134–150 μm) and at the center (distance from edge: 277–350 μm) of the colony. (J) Images of GFP-β-catenin hESCs at 46 h post treatment with either BMP4 (control) or BMP4 and SB. Average nonmembrane β-catenin levels as a function of radial position at 46 h. Error bars represent standard error. *N* ≥ 6. Underlying data can be found in S5 Data. BMP, Bone Morphogenic Protein; GFP, Green Fluorescent Protein; hESC, human embryonic stem cell; LDN, LDN193189.

To examine whether the BMP→WNT→NODAL cascade is conserved in our system, we first inhibited BMP signaling by adding its inhibitor LDN193189 (LDN) [57] at the time of BMP treatment and quantified the resultant WNT and NODAL signaling. We verified the function of LDN by immunostaining and found a near complete loss of pSMAD1/5/8, the key signal transducer of BMP signaling, within 5 h of LDN treatment, the earliest time point we measured (S6A Fig). In the absence of BMP signaling, WNT signaling shows a slight increase at the colony edge (Figs 5A, 5B and S6B, S9 Movie), and NODAL also remains at the colony edge (S6D Fig), indicating that BMP signaling is necessary to initiate WNT and NODAL waves. Addition of the WNT secretion inhibitor IWP2 at the time of BMP4 treatment also restricts NODAL signaling to the colony edge (S6D Fig), indicating that WNT signaling acts downstream of BMP to initiate the NODAL wave. NODAL signaling is also restricted to the colony edge in *NODAL* −/− cells (S6D Fig), suggesting that NODAL wave is a result of NODAL secreted by cells, while NODAL signaling at the colony edge is due to TGFβ1 in mTeSR1 (S1C Fig). Thus, the previously published BMP→WNT→NODAL cascade is also active in our gastrulation assay.

### BMP signaling controls the spatial extent and absolute levels of WNT signaling

Our previous work has shown that BMP is first active throughout the colony but becomes restricted to the colony edge around 12 h after treatment, and this pattern is then stable for the next 36 h [10]. To evaluate whether continuous BMP signaling is required for inward movement of WNT signaling, we inhibited BMP signaling using LDN during differentiation of micropatterned GFP-β-catenin hESCs at 2 different time points—11 h and 23 h post BMP treatment—and recorded the resulting WNT signaling by time-lapse imaging.

Quantification of WNT signaling dynamics shows that the final signaling levels are lower than the control sample and the signaling peak is shifted closer to the colony edge. WNT signaling movement, as reflected by the domain of active signaling, is also constrained (Fig 5A–5D, S10–S12 Movies). Thus, BMP signaling controls all aspects of WNT signaling, and therefore continuous BMP signaling is necessary to increase the levels of WNT signaling and to expand its active domain toward the colony center.

### WNT secretion controls the levels but not spatial extent of WNT signaling

Although WNT signaling levels depend on BMP signaling, the peak value of WNT signaling continues to increase after BMP signaling is inhibited (Fig 5B and 5C), indicating that WNT signaling is able to sustain itself when BMP is inhibited. To test whether continuous WNT secretion is required to maintain WNT signaling, we inhibited WNT secretion using IWP2 during differentiation of micropatterned GFP-β-catenin hESCs at 2 different time points—15 h and 30 h post BMP treatment—and recorded the resulting WNT signaling by time-lapse imaging.

Inhibition of WNT secretion lowers peak WNT signaling levels in a time-dependent manner, with inhibition at the earliest time point giving the lowest peak signaling, indicating that continuous WNT secretion is necessary to achieve high WNT signaling levels (Fig 5E–5G, S13–S15 Movies). Surprisingly, despite a reduction in signaling levels, the spatial dynamics of WNT signaling in the colony are similar: WNT activity first increases in a ring-like domain and then moves toward the colony center (Fig 5H). The inward movement happens at the same rate in all conditions as indicated by the movement of WNT signaling fronts (Fig 5I). This suggests that the movement of the wave of WNT signaling is independent of secretion of new WNT proteins and may rely on diffusive or active extracellular movement of the WNT proteins produced near the colony edge. Thus, WNT secretion after 15 h is required to increase the total levels, but not the spatial extent of WNT activity.

### NODAL signaling controls the spatial extent but not absolute levels of WNT signaling

Finally, we evaluated whether NODAL signaling, which lies downstream of WNT in the BMP→WNT→NODAL cascade, has an effect on WNT signaling activity by adding SB at the beginning of time-lapse imaging of BMP-treated micropatterned GFP-β-catenin cells. Surprisingly, NODAL inhibition constrains the peak of WNT signaling to the colony edge, similar to BMP inhibition (Fig 5J). However, unlike BMP inhibition, the peak WNT signaling levels are unaffected by NODAL inhibition (Fig 5J), suggesting that NODAL controls the movement but not the final levels of WNT signaling. Taken together, these results suggest that BMP signaling maintains WNT signaling levels by continuous production of new WNT ligands at the colony edge, and BMP and NODAL signaling synergize to control the inward movement of WNT signaling activity.

### NODAL signaling moves inward independently of BMP and WNT

**Fig 6:**
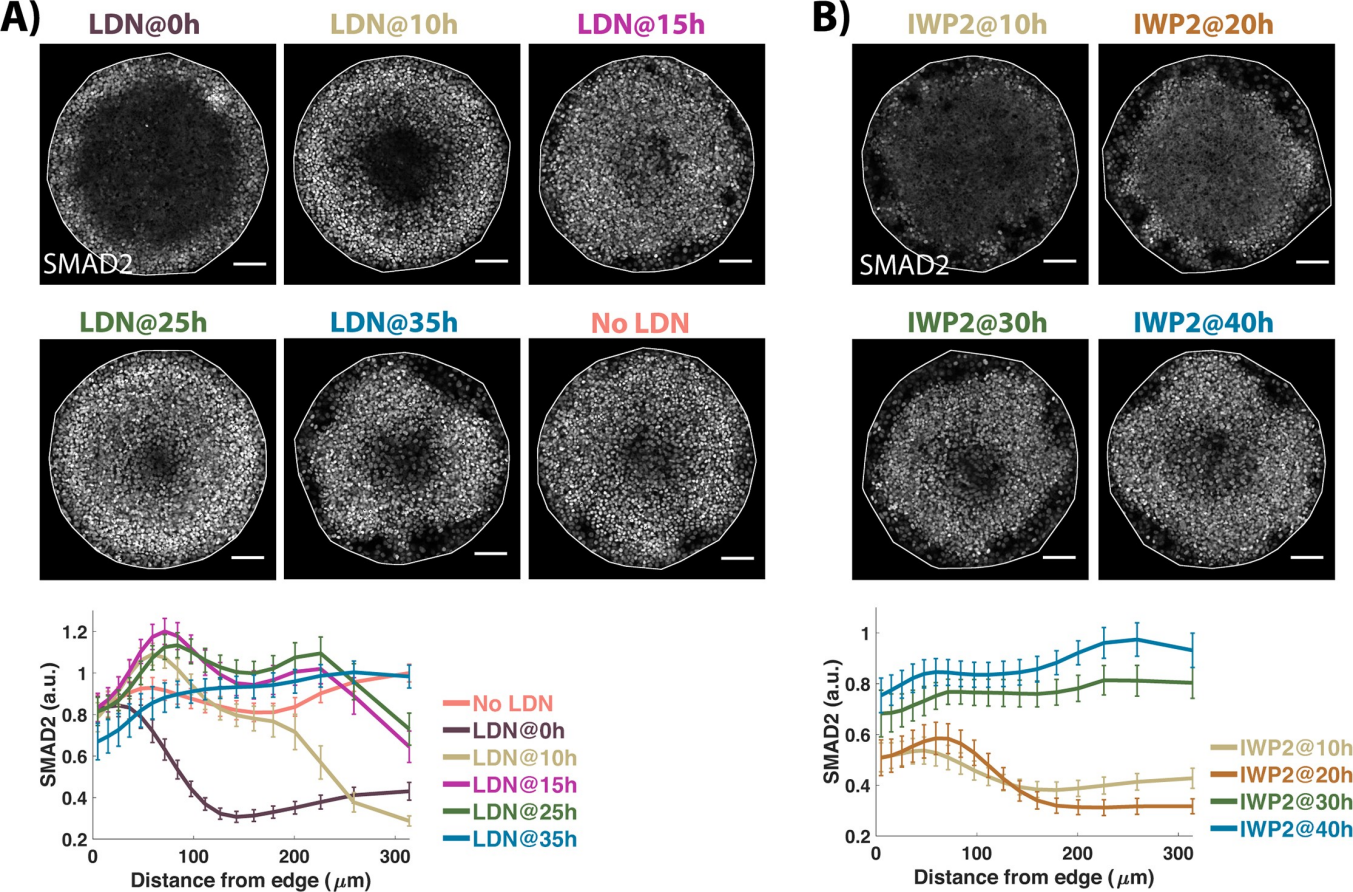
NODAL signaling moves inward independently of BMP and WNT. Images of samples immunostained for SMAD2/3 after 44 h of BMP treatment. The time between BMP4 and inhibitor (LDN or IWP2) addition is indicated above the image. No chemical inhibitor was added in the No-LDN sample. Quantification represents average nuclear intensities of indicated markers normalized to DAPI as a function of radial position. Error bars represent standard error. *N* ≥ 10. Scale bar = 100 μm. Underlying data can be found in S6 Data. BMP, Bone Morphogenic Protein; LDN, LDN193189.

Next, we examined the effects of BMP and WNT on NODAL signaling by inhibiting the signals at different time points during the gastrulation assay and quantifying the resultant NODAL signaling by immunostaining for SMAD2/3, the signal transducer of NODAL signaling. Upon inhibition of BMP signaling by LDN at 10 h, the front of active SMAD2 stops halfway to the center, but BMP signaling inhibition at or beyond 15 h does not prevent active SMAD2 from reaching the colony center, indicating that continuous BMP4 signaling is not required for the inward movement of NODAL signaling (Fig 6A). Interestingly, when LDN is added at 15 h, it strengthens the NODAL signaling at the colony border, indicating that prolonged BMP signaling at the border down-regulates NODAL signaling. Thus, 15 h of BMP signaling is sufficient for maximal activation of NODAL, and further BMP signaling primarily down-regulates NODAL at the colony edge. This is in contrast to WNT signaling, which—even after 23 h of BMP signaling—is constrained to the colony edge (Fig 5B), suggesting that, following its initiation by WNT, the NODAL signaling wave propagates independently of WNT signaling.

To determine when NODAL becomes independent of WNT signaling, we inhibited WNT secretion at different time points post BMP treatment by adding IWP2. We observed a binary effect on the movement of NODAL signaling. Inhibition in the first 20 h completely prevents the inward movement of NODAL signaling (Fig 6B). In these conditions, activity is restricted to the colony edges and is comparable to that observed in *NODAL−/−* cells indicating that early WNT inhibition abolishes the effects of paracrine NODAL (Figs 6B and S6D). Secretion inhibition at 30 h and beyond has no effect on inward movement of NODAL signaling activity, whereas at 25 h, we observe a mixture of these two phenotypes (Figs 6B and S5E). Thus, the NODAL wave is initiated between 20 and 30 h, consistent with our previous live cell measurements of signaling dynamics [10], and rapidly becomes independent of WNT signaling.

### WNT enhances, and NODAL inhibits, BMP signaling levels at colony edge

**Fig 7:**
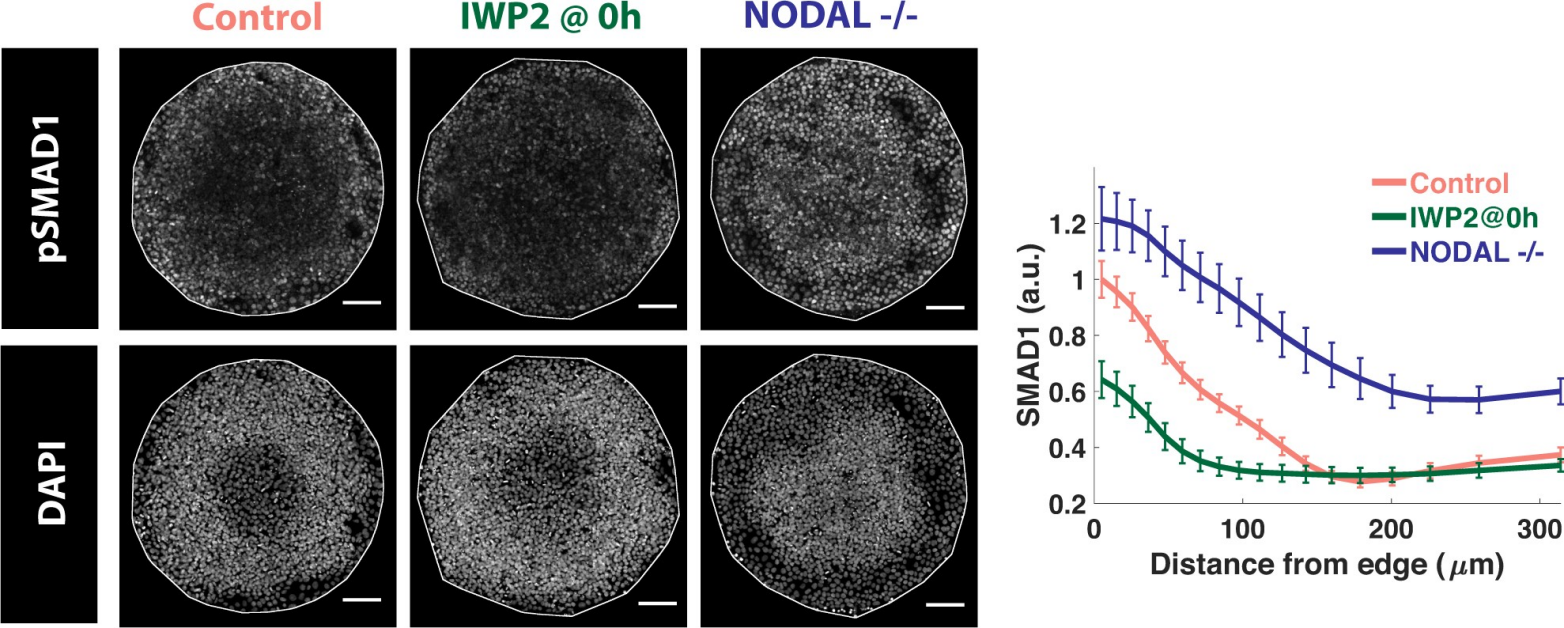
WNT enhances and NODAL inhibits BMP signaling at colony edge. Images of samples immunostained for the indicated markers after 44 h of treatment in different conditions: control (wild-type) hESCs and *NODAL*−/− cells were treated with 50 ng/ml BMP4. The sample labelled as IWP2 at 0 h represents wild-type cells treated with 50 ng/ml BMP and 5 μM IWP2. Quantification represents intensity levels of indicated markers normalized to DAPI, averaged at different positions along the colony radii. Error bars represent standard error of the mean. *N* ≥ 10. Scale bar = 100 μm. Underlying data can be found in S7 Data. BMP, Bone Morphogenic Protein; hESC, human embryonic stem cell.

We next evaluated whether WNT and NODAL signaling have any feedback on BMP signaling in the gastrulation assay by either adding IWP2 throughout or by using *NODAL−/−* cells and immunostaining for pSMAD1/5/8, the signal transducer of BMP signaling. These analyses revealed that WNT and NODAL have opposite effects on BMP signaling: BMP signaling is decreased by WNT inhibition and increased by NODAL inhibition (Fig 7), suggesting that WNT enhances while NODAL inhibits BMP signaling at the colony edge. Interestingly, NODAL inhibition also increased BMP signaling at the colony center, indicating that the sustained restriction of BMP signaling to the colony edge depends on NODAL signaling.

Taken together, these results map the influence of interactions in the BMP→WNT→NODAL cascade on signaling dynamics of the 3 pathways and reveal that the cascade is more interconnected than reported previously [1,33], with feedback from downstream signals in the cascade.

### Duration of BMP signaling correlates with extra-embryonic differentiation at colony edge

After characterizing signaling dynamics and determining the importance of interactions between BMP, WNT, and NODAL signaling pathways to achieve those dynamics, we evaluated which features of signaling dynamics are important for cell fate decisions. Our RNA-seq analyses suggest that extra-embryonic differentiation at the colony edge is driven by BMP signaling, independent of WNT and NODAL (Fig 2). Thus, the modulation of BMP signaling levels by WNT and NODAL as shown above (Fig 7) has no consequence for extra-embryonic differentiation at the colony edge, although it might explain the modulation of CDX2 protein levels in edge cells by WNT and NODAL inhibition (Fig 2A).

**Fig 8:**
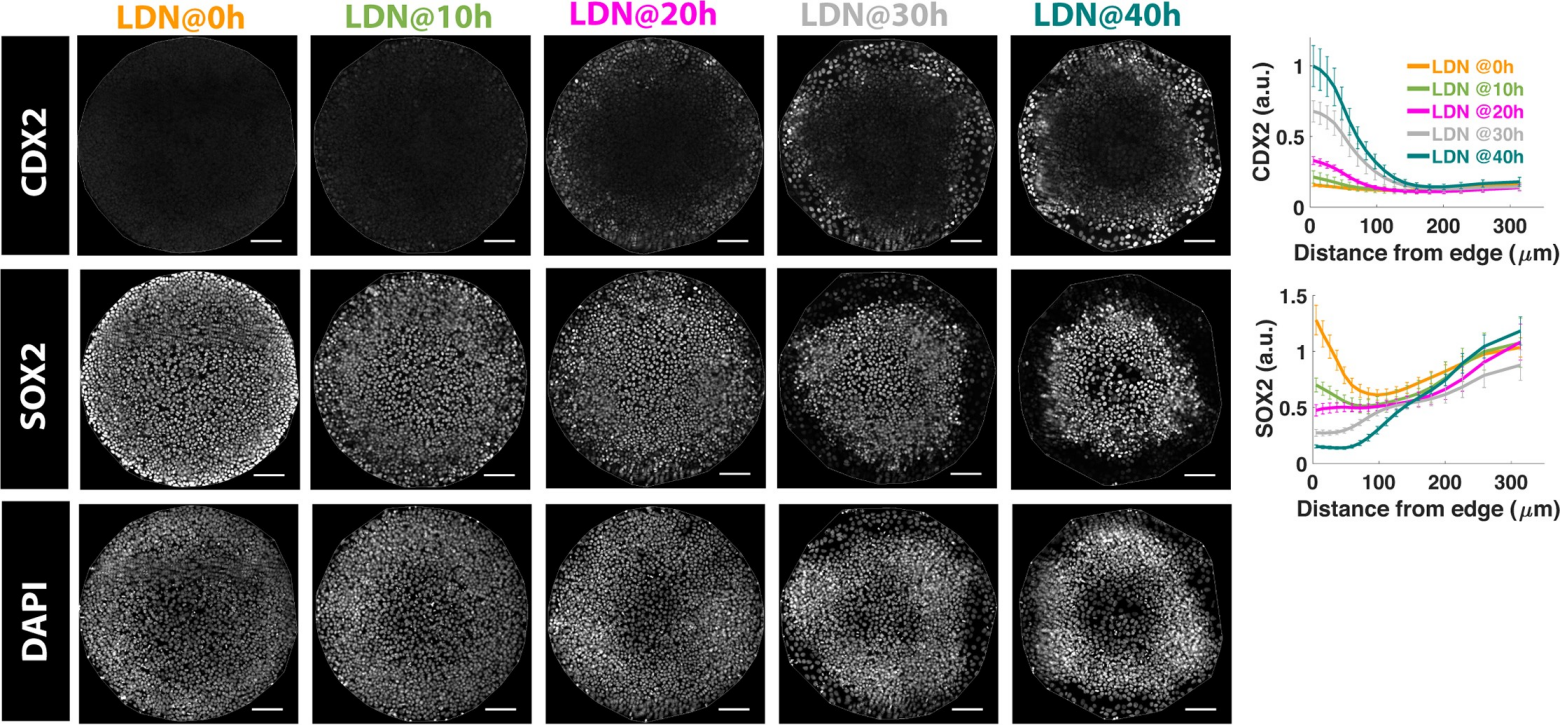
Duration of BMP signaling controls extra-embryonic differentiation at colony edge. Images of samples immunostained for fate markers after 44 h of BMP treatment. The time between BMP4 and LDN addition is indicated above the image. Quantification represents average nuclear intensities of indicated markers normalized to DAPI as a function of radial position. Error bars represent standard error. *N* ≥ 10. Scale bar = 100 μm. Underlying data can be found in S8 Data. BMP, Bone Morphogenic Protein.

Our previous results suggest that the duration of BMP signaling correlates with extra-embryonic CDX2+ differentiation under standard culture conditions [58]. Consistent with this, inhibition of BMP signaling in micropatterned colonies by LDN in the first 10 h leads to a complete loss of CDX2+ cells at the colony edges. Allowing BMP signaling for 20 h or more yields a CDX2+ population at the colony edges with longer durations of signaling yielding more CDX2 expression (Fig 8). Thus, after a minimum of 20 h, CDX2 is up-regulated in a graded manner by increasing BMP signaling duration.

### Continuous WNT and NODAL signaling synergize to achieve maximal mesodermal differentiation

Our results suggest that WNT signaling initiates, and NODAL signaling enhances, mesodermal differentiation (Fig 1A). Although the spatial dynamics of WNT and NODAL are similar, they respond very differently to perturbation of upstream signaling (Figs 5 and 6). To better understand how this network of signaling interactions specifies the region of mesodermal differentiation, we evaluated which features of signaling correlate with mesodermal differentiation when the signaling network is perturbed.

**Fig 9:**
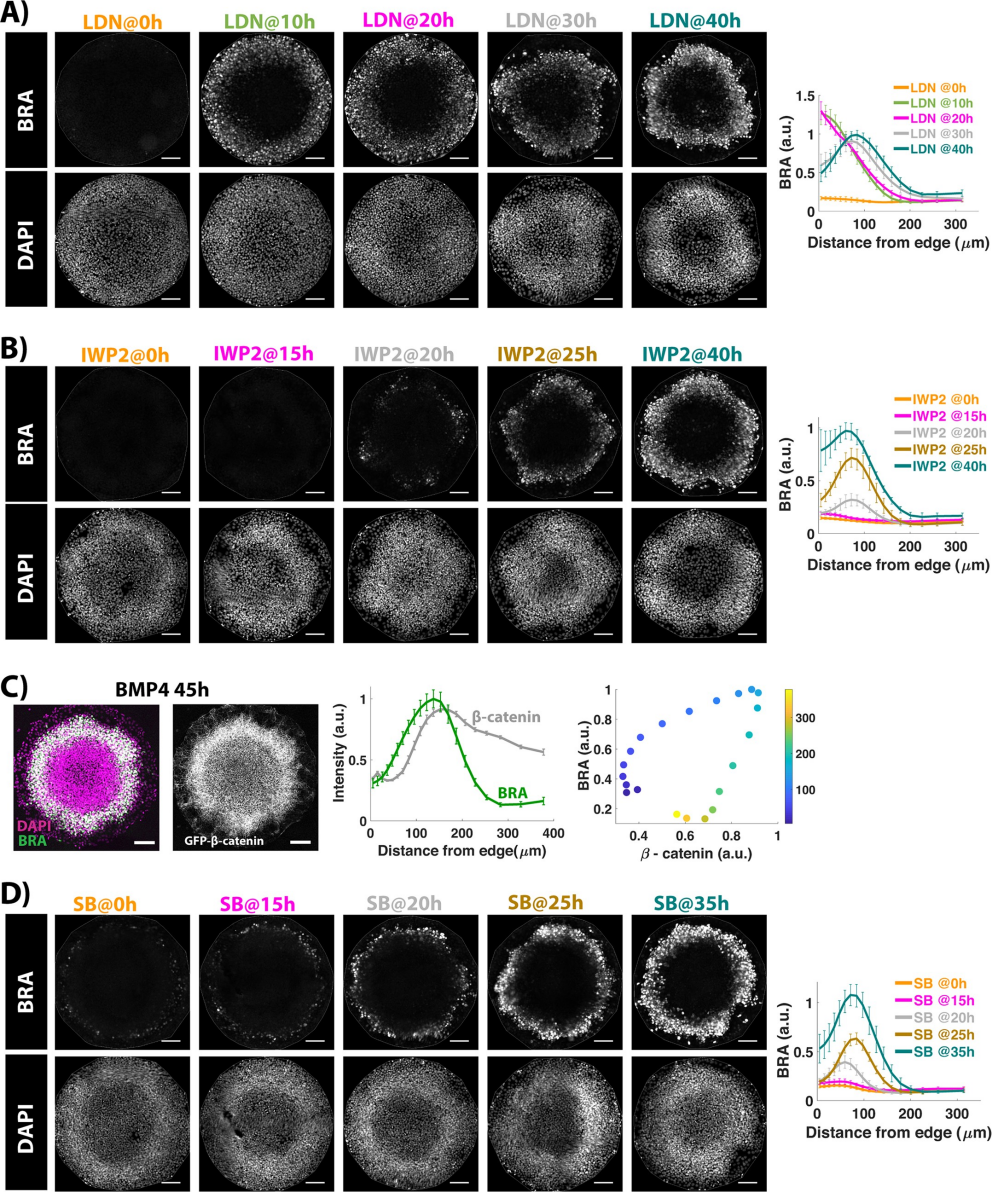
Continuous WNT and NODAL signaling synergize to achieve maximal mesodermal differentiation. (A, B, D) Images of samples immunostained for fate markers after 44 h of BMP treatment. The time between BMP4 and chemical inhibitor (LDN/IWP2/SB) addition is indicated above the image. Quantification represents average nuclear intensities of indicated markers normalized to DAPI as a function of radial position. Error bars represent standard error. *N* ≥ 10. (C) (Left image) Image of a colony immunostained for BRA and DAPI after 47 h time-lapse imaging of BMP-treated GFP-β-catenin hESCs. (Right image) Snapshot from time-lapse imaging for the same colony at 47 h. (Left plot) Quantification represents average intensity levels for BRA and nonmembrane β-catenin at 47 h, normalized by DAPI, plotted as a function of radial position. Error bars represent standard error of the mean. *N* = 9. (Right plot) Average BRA intensity levels as a function of β-catenin levels, color-coded by distance from the colony edge (μm). Scale bar: 100 μm. Underlying data can be found in S9 Data. BMP4, Bone Morphogenic Protein; BRA, BRACHYURY; GFP, Green Fluorescent Protein; hESC, human embryonic stem cell.

When the BMP, WNT, and NODAL signaling network is intact, WNT signaling continuously increases and eventually peaks in a ring-like domain that lies close to the mesodermal peak (Fig 3C and 3D). Early inhibition of BMP leads to an outward shift of peak WNT signaling and a subsequent corresponding outward shift in the mesodermal territory (Figs 5B, 9A and S7D), suggesting that mesodermal differentiation is closely linked to high WNT signaling. Consistent with this, varying the duration of WNT secretion by adding IWP2 at different time points increases mesodermal differentiation in a time-dependent manner, with the longest duration giving the maximal peak mesodermal differentiation (Fig 9B). However, the same levels of peak WNT signaling (Fig 5B and 5C: LDN at 11 h; Fig 5F and 5G: IWP2 at 15 h) can result in either maximal peak mesodermal differentiation (Fig 9A, LDN at 10 h) or no mesodermal differentiation (Fig 9B, IWP2 at 15 h). Similarly, within a given condition (BMP treatment for 45 h), the same WNT signaling levels at the inner and outer edge give 2 very distinct levels of mesodermal differentiation, as indicated by the average radial intensities (Fig 9C). These results suggest that there is no one-to-one mapping between WNT signaling levels and mesoderm differentiation, and the WNT signaling threshold for mesoderm differentiation varies depending upon the state of other signals.

Similar to WNT signaling, NODAL signaling activity does not spatially map to mesoderm differentiation, as it covers the entire colony at steady state (Fig 6A, No LDN). Thus, mesoderm differentiation does not depend on a particular threshold of one signaling pathway. However, modulating the duration of NODAL signaling by adding SB at different time points has a similar effect as IWP2 addition (Fig 9D), with the longest duration of NODAL signaling giving the maximal peak mesodermal differentiation suggesting that continuous signaling via WNT and NODAL synergize to achieve maximal mesodermal differentiation.

### A simple mathematical model recapitulates signaling dynamics and predicts cell fate patterning

Our data suggest that BMP signaling initiates waves of paracrine WNT and NODAL signaling that display similar spatial dynamics but distinct regulation by upstream signals. Here, we translate these interactions into a three-component mathematical model that recapitulates signaling dynamics under chemical and geometric perturbations.

**Fig 10:**
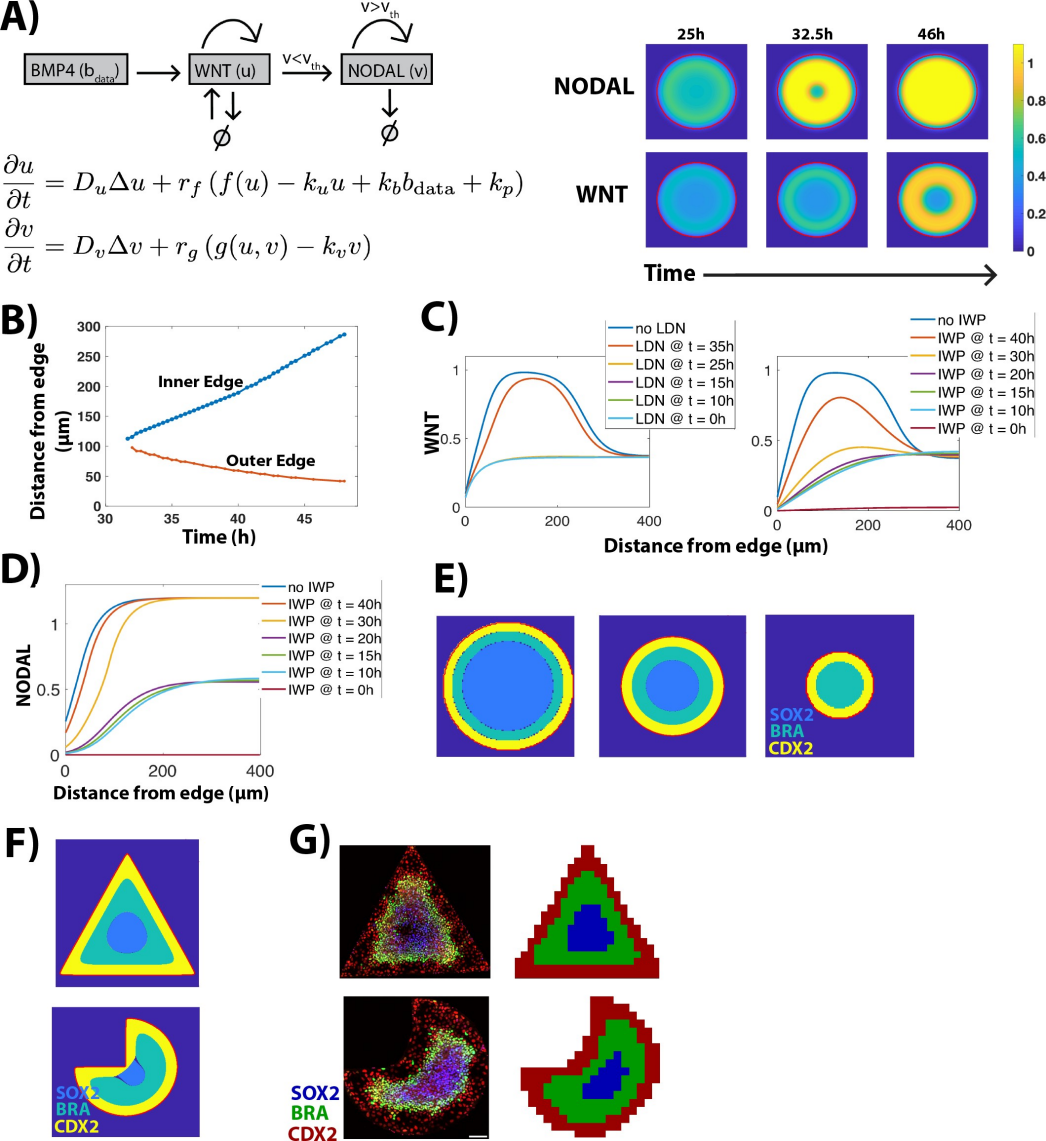
A simple mathematical model recapitulates signaling dynamics and predicts cell fate patterning. (A) (Left) Model equations. *u* and *v* represent WNT and NODAL signaling, respectively (parameter descriptions and values in S2 Model). (Right) Time evolution of WNT and NODAL to steady state. (B) The position of inner and outer edge of simulated WNT signaling levels as a function of time. (C) WNT levels at steady state (46 h) as a function of edge distance for different simulations. LDN addition was simulated by setting WNT’s dependence on BMP to 0 at indicated time points. IWP2 addition was simulated by setting autoactivation of WNT to 0 at indicated time points. (D) NODAL levels at steady state (46 h) for different simulations. (E, F) Fates assigned by the model on colonies of different sizes (E), shapes (F). (G) hESCs immunostained for CDX2, BRA, SOX2 after 44 h of BMP treatment on triangular and pacman colonies. Immunostaining data from *n* = 18 colonies were used to calculate average fate territory maps shown adjacent to image. Scale bar = 100 μm. Underlying data can be found in S10 Data. BMP, Bone Morphogenic Protein; BRA, BRACHYURY; hESC, human embryonic stem cell; LDN, LDN193189.

The model represents interactions between BMP, WNT, and NODAL signaling as a system of partial differential equations. For simplicity, we only include the sequential effect of BMP on WNT and of WNT on NODAL and do not include feedback on upstream signals. We further assume basal and autoactivation of WNT signaling, to take into account the fact that WNT signaling reaches a nonzero value following early termination of BMP signaling (Fig 5A–5D). Similar to WNT, NODAL signaling is also autoactivated but only when it crosses a certain threshold value (v_th_). Below this threshold, NODAL signaling is activated only by WNT signaling. This assumption takes into account the fact that NODAL wave becomes independent of WNT signaling following its activation (Fig 6). To simulate the model, we use our experimental data on BMP signaling dynamics [10] as an input (S16 Movie) to the reaction-diffusion system composed of WNT and NODAL signaling (Fig 10A, S2 Model). Thus, the model simulates the time evolution of WNT and NODAL signaling in response to experimentally determined dynamics of BMP signaling.

The model recapitulates the inward movement of both WNT and NODAL signaling, with NODAL moving in faster than WNT. While NODAL signaling displays a uniformly high activity everywhere in the colony except the colony edges, WNT signaling forms a ring-like domain of high activity at steady state (Fig 10A, S17 and S18 Movies). The front of active WNT signaling moves inward at a constant rate (Fig 10B), as observed experimentally.

Next, we examined whether the model can reproduce the effects of inhibiting upstream signals at varying time points on WNT and NODAL signaling dynamics. Early inhibition of BMP signaling reduces WNT signaling levels as observed experimentally. However, the simulations do not reproduce movement of peak WNT signaling toward the colony edge (Fig 10C, Fig 5A–5D). As cells at the colony edge respond to BMP but do not show high WNT signaling (Figs 7, 3C and 3D), it is likely that WNT signaling is repressed by BMP in the edge cells. This interaction is not incorporated in the model, and a more complex model would be required to mimic this behavior. In contrast, the effect of inhibition of WNT secretion on both WNT and NODAL signaling in the model is consistent with experiments. WNT signaling levels reduce, but the position of peak WNT signaling remains unaffected (Fig 10C). WNT secretion inhibition in the first 20 h abolishes the NODAL wave but does not affect its movement post 20 h (Fig 10D). Thus, this simple model is capable of reproducing most experimental data on time-dependent perturbations to signaling.

Finally, we employed a heuristic approach to simulate the domain of each cell fate on the circular colonies as a combinatorial function of BMP, WNT, and NODAL. To do this, we compared the simulation dynamics on circular colonies with the experimentally determined fate pattern and chose simple phenomenological functions that can implement this mapping (S2 Model). The model correctly predicted the loss of center fate in smaller circular colonies as was observed previously (Fig 10E [21]). We simulated the signaling dynamics on other shapes and applied these functions without modification to the simulated signaling dynamics in order to predict the results of changing the geometry of the colony. We found an inward expansion of fates at the corners of noncircular colonies (Fig 10F). We tested this prediction experimentally by performing the micropatterning assay on triangular and pacman-shaped colonies. We then analyzed colonies with similar cell densities across all shapes (S8B and S8C Fig). As predicted by the model, both triangle and pacman-shaped colonies display an inward expansion of fates at the corners of the colony (Figs 10F and S8D). Thus, the model is capable of predicting fate patterns on shapes, which were not considered in its development.

## Discussion

Genetic and biochemical studies in the mouse embryo over the past decades have revealed that BMP, WNT, and NODAL signaling pathways are integral to gastrulation. As WNT and NODAL ligands are expressed at the posterior end of the mouse embryo where the primitive streak forms, the data support a model in which a signaling gradient of high WNT and NODAL signaling activities drives primitive streak formation [1]. However, a gradient in WNT and NODAL signaling activities during gastrulation has never been directly observed, and the available in vivo data are indirect and open to alternate interpretations.

Here, using an in vitro human gastruloid model, we quantitatively examine WNT and NODAL signaling activities during gastrulation. Our data suggest that the simplest picture consistent with previous data—that stable signaling gradients underlie primitive streak formation—is likely incorrect. Instead, dynamic waves of WNT and NODAL, which have no stable pattern at steady state, underlie primitive streak formation. As with any new model derived from stem cell studies, in vivo validation will be required to say how closely these waves and their interpretation are mirrored during development.

In the gastruloid model, BMP signaling initiates waves of WNT and NODAL signaling that move toward the colony center at constant rates. Using mathematical modeling, we show that this signaling behavior lies outside the Turing instability regime that generates spatial gradients of signaling activities. Instead, the wave-like behavior of WNT and NODAL arises due to initial activation of WNT and NODAL at the colony boundary and subsequent autoactivation and diffusion within the colony. The final signaling state of the system is homogeneous, and low signaling levels at the colony edge arise due to diffusive loss of signals from the boundary. This result shows that fate patterning can be achieved in a system with homogenous steady state signaling and, by extension, rules out a model in which fates are determined by thresholds in a single morphogen pathway.

Reaction-diffusion–based Turing systems are theoretically capable of achieving pattern formation from an initially homogeneous state and have been experimentally shown to be active in many biological processes [13–15, 18–20]. Within human gastruloids themselves, the dynamics of BMP and its inhibitor NOGGIN was proposed to be a Turing system [22]. However, the fact that exogenous BMP dose can modulate the length scale of the BMP signaling pattern within the colony argues against this hypothesis, because this would be not be the case for a self-organized pattern driven by autoactivation and inhibition. Additional tests, for example, determining whether the BMP-NOGGIN network can break symmetry and establish the BMP signaling pattern in the absence of a colony boundary, will be necessary to determine whether BMP-NOGGIN can create a diffusion-driven Turing instability [59, 60].

Our results also reveal the complexity underlying the previously proposed linear cascade of BMP→WNT→NODAL signaling. Although WNT and NODAL display similar spatial dynamics, they display distinct temporal regulation by upstream signals. While the movement of WNT signaling depends on BMP, NODAL signaling—once initiated—moves inward independently of upstream BMP and WNT signaling (Figs 5 and 6). We also show that WNT signaling positively feeds back on BMP signaling, whereas NODAL signaling has the opposite effect (Fig 7).

Taken together, our data suggest that fate patterning in gastruloids happens in 2 phases. In the first phase (1–12 h post treatment), BMP signaling is first widespread and then restricted to the colony edges through receptor localization and NOGGIN production [10, 32]. The position of prolonged BMP signaling sets the spatial range of differentiation to an extra-embryonic fate. In the second phase, dynamic waves of WNT and NODAL signaling move through the colony and set the location of mesendodermal fates. Because the WNT wave depends on BMP (Fig 5), and WNT and NODAL also influence BMP signaling (Fig 7), the 2 phases appear to be interdependent. The precise molecular mechanisms controlling this interdependence will require further investigation.

A similar dynamic wave of WNT signaling was shown to underlie primitive streak formation in another model of human gastruloids [61]. There, it was shown that WNT-treated micropatterned hESCs exhibit a prepattern in which low E-cadherin at the colony edge allows a strong response to WNT signaling in edge cells. WNT then combines with NODAL signaling to initiate primitive streak differentiation and EMT, which down-regulates E-cadherin in the neighboring cells and allows them to respond to the exogenous WNT. Thus, when micropatterned colonies are stimulated with WNT, a wave of EMT propagates inward and underlies the apparent movement of the WNT signal in the colony [61]. In contrast, here, we show that in BMP-treated micropatterned hESCs, the inward expansion of WNT signaling activity does not start at the colony edge (Fig 3D–3F) where E-cadherin would be low, suggesting that a similar E-cadherin–mediated mechanism is unlikely to be involved. Furthermore, in WNT-treated micropatterned colonies, the wave of signaling is entirely due to exogenous WNT and is not blocked by inhibition of WNT secretion, whereas in BMP-treated colonies, WNT signaling and mesoderm differentiation are due to endogenous signals (Fig 1). Thus, although the dynamics of WNT movement are qualitatively similar, the mechanisms are distinct: one involves propagation of WNT ligands, while the other involves constant signal and a propagation of the competence to respond to that signal.

What controls the inward movement of WNT activity in BMP-treated gastruloids? Absence of cell movement (Fig 4) implies that either WNT ligands move at long distances through colonies or cells transfer WNT ligands in a relay-like manner such that signaling in one cell causes that cell to produce WNT ligands that activate signaling in its neighboring cells, and the activity continues. Our data suggest that it is likely to be the former. WNT signaling activity continues to move inward even after inhibition of WNT secretion (Fig 5I IWP2 at 15 h), indicating that the wave of WNT signaling results from the inward movement of WNT produced at the colony edges prior to secretion inhibition. Inhibiting WNT secretion prevents autoactivation of the pathway as these ligands move inward, thus lowering the magnitude of the response but leaving the range of signaling unaffected. If true, this would indicate that WNT proteins have the potential to activate signaling at long range, as recently observed in the *Caenorhabditis elegans* embryo [62] and in contrast with the short-range activation in the mammalian intestinal crypt [63]. The linear scaling in time of the movement of WNT signaling in the colony, even in the absence of secretion of new WNT ligands, suggests that WNT ligands do not move by passive diffusion, as this would be expected to scale with the square root of time. Interestingly, the movement of WNT activity requires both BMP and NODAL signaling (Fig 5). One plausible hypothesis is that the combined effect of BMP and NODAL signaling is required for this active movement of WNT ligands. A second is that these signals are required for the competence of cells in the colony to respond to WNT ligands. Future studies will be needed to dissect the mechanisms underlying the movement of both WNT and NODAL signaling activities.

Our data also present evidence for the trophectodermal cell fate of BMP-treated CDX2+BRA− hESCs and argue against their identification as extra-embryonic mesodermal cells. Because hESCs are thought to represent the epiblast, a cell state that does not contribute to the trophoblast population in vivo, the fact that trophoblast cell types can be derived from hESCs has remained controversial. One study suggested that these cells represent extra-embryonic mesoderm, and not trophoblast cells. They showed that BMP treatment induces co-expression of BRA and CDX2, where BRA precedes and is necessary for CDX2 expression, indicating that these cells have a mesodermal origin and likely represent extra-embryonic mesoderm cells that are derived from the primitive streak in the mouse embryo [44,51]. Several lines of investigation argue against this conclusion. First, inhibiting WNT or NODAL signaling—which drastically reduces mesodermal differentiation—does not affect this population (Figs 1 and 2). Second, by systematically comparing the transcriptomes of BMP-treated CDX2+ cells with in vivo human cells, we show that these BMP-treated hESCs are transcriptionally similar to the trophoblast lineage and express many early trophectoderm markers, including GATA3, TFAP2C, and CDX2. They also express some markers seen in CT cells of week 6 placenta, indicating they likely belong to a developmental stage in between week 2 and week 6 (Fig 2). Third, unlike the mouse, extra-embryonic mesoderm in primates is present prior to the primitive streak [49–51] and thus may not express primitive streak markers like BRA. In the monkey embryo, extra-embryonic mesoderm cells express markers of PE—*GATA4* and *GATA6*—and are transcriptionally similar to PE cells, indicating a PE origin [52]. Given the similarity between human and monkey embryos, it is likely that human extra-embryonic mesodermal cells have a similar PE origin, thus challenging the claim made in the previous study [44]. We found that BMP-treated hESCs do not express PE markers *GATA4* and *GATA6* and are therefore unlikely to represent extra-embryonic mesoderm.

In the gastruloid model, we show that the duration of BMP signaling controls trophectodermal differentiation at the colony edge (Fig 8), consistent with our previous observations [58]. By measuring signaling duration, cells are effectively measuring the integrated levels of BMP signaling. How cells perform this computation to differentiate toward the trophectodermal cell fate remains an open question.

Finally, we show that although WNT and NODAL signaling waves control mesodermal differentiation, both waves move further toward the center of the colony than the ring of mesodermal differentiation (Figs 3,6 and 9), suggesting that mesodermal differentiation does not depend on a particular signaling level of either WNT or NODAL. What, then, defines the boundaries of mesodermal ring? Given that both WNT and NODAL fronts move inward at different speeds, with NODAL moving faster than WNT ([10], Fig 3F), one hypothesis is that the time interval between the WNT and NODAL activation is a key parameter in determining mesoderm differentiation, consistent with a recent study on NODAL dynamics [11]. Another hypothesis is that because BMP signaling is activated homogeneously before being restricted to the edge [10], the timing between BMP signaling and the WNT and NODAL waves may determine the position of mesodermal differentiation. A third hypothesis is that signaling thresholds in multiple pathways, possibly evaluated in different time intervals, govern mesoderm differentiation. It is important to note that these hypotheses are not mutually exclusive, and all suggest that it is the combinatorial function of multiple signals rather than the threshold of a particular signal that controls mesoderm differentiation.

Ethical challenges limit research on human embryos in vivo, making gastruloids a valuable tool to investigate human gastrulation. We experimentally and theoretically show that in this model mesoderm patterning is not due to an underlying spatial signaling pattern. As recent technical advances [64] make it possible to image gastrulating mouse embryos with cellular resolution, it will soon be possible to test whether a similar wave-like behavior of WNT and NODAL signaling activities precedes BRA expression. In the vertebrate neural tube, mutually inhibitory interactions in the gene regulatory network that decodes Sonic hedgehog signaling are integral to establishing spatial fate patterns [65]. It will be interesting to decipher the gene regulatory network that decodes position and thus cell fates from the dynamics of signaling events during the self-organized fate patterning of human gastruloids.

## Materials and methods

### Experimental system

#### Cell lines

Experiments were performed using ESI017 (obtained from ESI BIO, RRID: CVCL_B854, XX) hESC line. For WNT signaling dynamics, the ESI017 GFP-β-catenin cell line as described in [55] was used. For cell tracking experiments, transgenic RUES2 cell line (RUES VENUS:H2B) (a gift from from Ali Brivanlou, Rockefeller, RRID: CVCL_B810, XX) was used.

### Routine cell culture

All cells were grown in the chemically defined medium mTeSR1 in tissue culture dishes and kept at 37°C, 5% CO_2_ as described in [58]. Cells were routinely passaged and checked for mycoplasma contamination also as described in [58].

### Micropatterning

Micropatterning experiments were performed on either micropatterned chips or 96-well micropatterned plates obtained from CYTOO (shapes, 96-well plate, circles). In both cases, hESCs were seeded onto micropatterned surfaces coated with 5 μg/ml Laminin-521 using the mTeSR1 protocol described in [34]. Following seeding, cells were either treated with 50 ng/ml BMP4 (gastrulation assay, control sample) and/or with reagents as described in the text.

### Immunostaining

Immunostaining followed standard protocols as described in [58]. Primary and secondary antibody were diluted in the blocking solution as described in [21, 58]. Dilutions are listed in the reagents table (Table 1).

**Table 1:** Resources table.

Reagent or Resource	Source	Identifier
***Antibodies***		
β-ACTIN Peroxidase C (1:5000)	Sigma-Aldrich, St. Louis, MO	Cat# A3854-200UL
BRA (1:300)	R&D Systems, Minneapolis, MN	Cat#AF2085
CDX2 (1:50)	Biogenex, Fremont, CA	Cat#MU392A
GATA3(1:100)	Thermo Fisher Scientific, Waltham, MA	Cat#PA1-101
HAND1(1:200)	R&D Systems	Cat#AF3168
ISL1(1:50)	Developmental Studies Hybridoma Bank, University of Iowa, Iowa City, IA	Cat#39.4D5
NANOG (1:100)	BD Biosciences, San Jose, CA	Cat#560482
NODAL mAB11 (1:200)	Harvey Lab, MD Anderson, Houston, TX	N/A
SOX17 (1:200)	R&D Systems	Cat#AF1924
phosphoSMAD1 (1:200)	Cell Signaling Technologies, Danvers, MA	Cat#13820
SOX2 (1:200)	Cell Signaling Technologies	Cat#5024S
SMAD2/3 (1:100)	BD Biosciences	Cat#610842
TFAP2A(1:50)	Developmental Studies Hybridoma Bank	Cat#3B5
***Bacterial Strain***		
5-alpha Competent *Escherichia coli*	New England Biolabs, Ipswich, MA	Cat# C2987H
***Chemicals*, *Peptides*, *and Recombinant Proteins***		
BMP4	Thermo Fisher Scientific	Cat#314BP050
CHIR 99021	MedChem Express, New York, USA	Cat#HY-10182
cOmplete Lysis-M	Sigma-Aldrich	Cat#04719956001
cloneR	STEMCELL Technologies, Cambridge, MA	Cat#05889
DAPI (4,6-diamidino-2-phenylindole, dihydrochloride)	Thermo Fisher Scientific	Cat#D1306
DL-Dithiothreitol	Sigma-Aldrich	Cat# D9163-5G
ECL Western Blotting Substrate_ Promega W1001	Promega, Madison, WI	Cat# PAW1001
GE Healthcare Amersham Hyperfilm ECL	Thermo Fisher Scientific	Cat# 45-001-508
IWP2	Stemgent, Cambridge, MA	Cat#04–0034
Laemmli Sample Buffer	Bio-Rad	Cat#1610737
LDN	Thermo Fisher Scientific	Cat#04-0074-02
mTeSR1	STEMCELL Technologies	Cat#85875
Dulbecco's PBS Without calcium and magnesium	Caisson Labs, Smithfield, UT	Cat# PBL01-6X500ML
Puromycin	Thermo Fisher Scientific	Cat#A1113803
ROCK inhibitor Y-27632	Thermo Fisher Scientific	Cat#50-175-998
SB	Stemgent	Cat#04-0010-05
***Critical Commercial Assays***		
DNeasy Blood and Tissue Kit	Qiagen, Germantown, MD	Cat#69504
HiSpeed Plasmid Midi Kit	Qiagen	Cat#12643
Invitrogen TOPO TA Cloning Kit for Sequencing, without competent cells	Thermo Fisher Scientific	Cat#450030
P3 Primary Cell 4D-Nucleofector X Kit L	Lonza, Texas, USA	Cat# V4XP-3024
***Experimental Models*: *Cell Lines***		
ESI-017	ESI BIO, Alameda, CA	RRID:CVCL_B854
ESI017 GFP-β-catenin	[55]	
RUES2	Ali Brivanlou, Rockefeller University, New York, NY	RRID:CVCL_B810
***Oligonucleotides***		
*NODAL*_sgRNA_Forward:- CACCGGGCCCACCAGGCGTGCAGA	Integrated DNA Technologies, New York, US	N/A
*NODAL*_sgRNA_Reverse:- AAACTCTGCACGCCTGGTGGGCCC	Integrated DNA Technologies	N/A
*NODAL* genomic DNA PCR amplification: *NODAL* 5UTR-seq-Fwd: 5′-TTGCAGCCTGAGTGGAGAGG-3′ *NODAL* gDNA_seq_Rev: 5′-AACCCACAGCACTTCCCGAGTC- 3′	Integrated DNA Technologies	N/A
*NODAL* sequencing *NODAL* KO_confirm_Fwd 1 AGCTTCCCCAGAGGGAGGAAAGG *NODAL* KO_confirm_Rev 1 TGCAGAAGGAAGGGCAGGCAGTG *NODAL* KO_confirm_Rev 2 AGCATGTACGCCAGAGGGGATGG	Integrated DNA Technologies	N/A
***Recombinant DNA***		
pSpCas9(BB)-2A-Puro (PX459)	Addgene, Cambridge, MA	Catalog#48139
PX459-sgRNA_*NODAL*	This paper	
***Software and Algorithms***		
Benchling		https://benchling.com/
ilastik	[66]	http://ilastik.org/
MATLAB		https://www.mathworks.com/products/matlab.html
Matlab scripts for quantifying, analyzing data, and running simulations	This paper	https://github.com/sc65/CellTracker https://github.com/sc65/pde_simulation

**Abbreviations:** BRA, BRACHYURY; LDN, LDN193189; SB, SB431542

### Creation of NODAL knockout (*NODAL−/−)* cells

We introduced a mutation in exon 1 of the *NODAL* gene in ESI017 hESCs using CRISPR-Cas9. A guide RNA (sgRNA) directed toward *NODAL* exon1 was designed using benchling. The single stranded oligonucleotides for sgRNA were

*NODAL*_sgRNA_Forward:- CACCGGGCCCACCAGGCGTGCAGA; *NODAL*_sgRNA_Reverse:- AAACTCTGCACGCCTGGTGGGCCC

These oligonucleotides were annealed and inserted into the PX459 vector through BbsI restriction sites using standard restriction and ligation protocols. The insertion was verified using DNA sequencing. DNA was nucleofected into 8 × 10^5^ hESCs using P3 Primary Cell 4D-Nucleofector X Kit L (Lonza). Following nucleofection, cells were transferred into mTeSR1 with 10 μM ROCK inhibitor. Cells were selected by adding 1 μg/ml Puromycin to the nucleofected cells the subsequent day. To increase survival of selected cells, mTeSR1 was supplemented with CloneR after 1 d of antibiotic selection. After 5–6 d in mTeSR1 and CloneR, single colonies were picked and transferred to a 24-well plate. Genomic DNA from selected cells was extracted using DNeasy Blood and Tissue Kit. The genomic region around the *NODAL* gene was PCR amplified using the following primers:

Forward primer: 5′-TTGCAGCCTGAGTGGAGAGG-3′;

Reverse primer: 5′-AACCCACAGCACTTCCCGAGTC- 3′.

The PCR product was cloned using the Invitrogen TOPO TA Cloning Kit, and the DNA from individual bacterial colonies was sent for DNA sequencing. Sequencing results from the clone used in this study showed the presence of only 2 distinct mutations (S2 Fig) on the *NODAL* genomic locus, suggesting the enrichment of a monoclone with a distinct mutation on each allele. The absence of functional NODAL protein was verified by western blot.

### Western blot

Wild-type ESI017 hESCs and *NODAL−/−* hESCs were treated with 10 μM WNT agonist CHIR99021[67] for 20 h, which activates the WNT-β catenin pathway and up-regulates *NODAL* [68]. After treatment, cells were washed with PBS and lysed with cOmplete Lysis-M solution. Following lysis, cells were mixed with 2× Laemmli Sample Buffer supplemented with 200 mM dithiothreitol. The samples were denatured by heat at 95°C for 5 min and loaded to 4%–20% Mini-PROTEAN TGX Precast Gel (Bio-Rad). After electrophoresis at 120 V for 90 to 120 min, the sample was transferred to polyvinyl difluoride (PVDF) membrane. The PVDF membranes were blocked in PBST with 5% nonfat milk. The primary antibodies (NODAL and beta actin) were dissolved in PBST with 2% nonfat milk and incubated on the membrane at 4°C overnight. The membrane was washed 3 times with DPBST (1× DPBS with 0.1% Tween 20). Horseradish peroxidase conjugated secondary antibodies were applied to the membrane and incubated at room temperature for 1 h. The membranes were washed 3 times with PBST, and the signal was detected by using ECL western-blotting substrate and Amersham Hyperfilm ECL.

### Imaging

#### Live cell imaging

For the cell tracking experiment (Fig 4), cells with a nuclear fluorescent marker (RUES2-VENUS-H2B) were mixed with unlabeled cells (ESI017) in the ratio 1:100, seeded onto to a micropatterned chip kept in a holder (CYTOO), and treated with 50 ng/ml BMP4 as described in [34]. Six colonies of 800 μm diameter were imaged with a 10X, NA 0.40 objective on an Olympus laser scanning confocal microscope; 5–8 z-slices were acquired for each position every 10 min from 20 h to 47.5 h post BMP4 treatment. For the cell sorting experiment (S5A Fig), single cell suspensions of ESI017-CFP-H2B cells were mixed with one of the other 3 cell types—ESI017-RFP-H2B, ESI017-RFP-H2B differentiated to CDX2+ extra-embryonic cells, or RUES-VENUS-H2B, in the ratio of 3:7, seeded onto an ibidi μ-slide in mTeSR1 with Rock inhibitor and imaged on Olympus Andor spinning disk confocal microscope with 20X, NA 0.75 objective, every 10 min for 23 h. For determining WNT signaling dynamics during differentiation (Fig 3C), GFP-β-catenin-hESCs were seeded and treated as above. Nine colonies of 800 μm diameter were imaged with a 20X, NA 0.75 objective on a laser scanning confocal microscope with 5 z-slices acquired per position every hour from 3 h to 47 h post BMP4 treatment. For comparing WNT signaling dynamics across multiple conditions (Fig 5), GFP-β-catenin-hESCs were seeded in multiple wells of a 96-well micropatterned plate (CYTOO), with one experimental condition per well. Colonies of 700 μm were imaged at 20X resolution, NA 0.75 on a spinning disk confocal microscope, with 5 z-slices acquired per position every 30 min from 3 h to 47 h post BMP4 treatment. Cells were treated with 200 nM LDN or 5 μM IWP2 at the indicated times. The number of colonies imaged for each condition were as follows: LDN at 0 h: 3; LDN at 11 h: 3; LDN at 23 h: 5; control with no LDN: 13; IWP2 at 15 h: 5; IWP2 at 30 h: 5; and control with no IWP2: 5—where time in each condition represents time post BMP4 treatment when the indicated reagent was added. For all the previously mentioned experiments, cells were maintained at 37°C and 5% CO_2_ during imaging.

#### Fixed cell imaging

Immunostaining data for signaling transducers pSMAD1 and SMAD2 (Figs 6 and 7) were acquired with a 20X, NA 0.75 objective on a laser scanning confocal microscope with >5 z-slices per position. Immunostaining data for all other markers were acquired by imaging entire fixed micropatterned chips and 96-well micropatterned plates using tiled acquisition with a 10X, NA 0.40 objective on an Olympus IX83 inverted epifluorescence microscope. For data visualization purposes, sample images for each marker were acquired using 20X resolution NA 0.75 on laser scanning confocal microscope. Raw images for immunostaining data in each main Fig correspond to images taken at 20X and average plots represent quantification of images taken at 10X.

### RNA-seq

The treatments used were as described in the main text with the following concentrations: BMP4 10 ng/ml, SB 10 μm, and IWP2 4 μm. Total RNA was collected with the Invitrogen RNAqueous Micro Kit. Processed RNA was stored at −80°C, and RNA integrity was checked by Nanodrop, agarose gel electrophoresis, and the Agilent 2100 system. Sequencing was performed by Novogene Co. using the Illumina paired-end 150 platform (HiSeq2500).

### Quantification and analyses

All experiments were performed at least twice with consistent results. The data and analyses in each figure belong to one experiment. Sample size was not predetermined, and no statistical tests were used to determine significance of results. Circular colonies with a nonradial cell density pattern at the end of 44 h of BMP4 treatment were excluded from analyses. The number of colonies included in each analysis (*N*) is mentioned in the Figure legends. For images taken at 20X magnification with multiple z-slices, background subtraction, maximum z projection and alignment were performed as described in [21]. Colony images obtained after alignment were analyzed as described in S9 Fig using custom-made MATLAB scripts.

For the cell tracking experiment, cells were tracked using the tracking workflow in Ilastik version 1.2.0 [66]. Of the 165 labeled cells in 6 colonies, at least one daughter cell of 84 cells was tracked correctly during the entire course of imaging. Cells that died, went out of focus, or were mistracked were excluded from analyses.

For RNA-seq data, raw sequence reads obtained from Illumina were aligned to the genome using the STAR-2.6.1d alignment software [69]. Human genome assembly file hg38, obtained from the UCSC website, was used to create the annotated genome for STAR mapping. STAR output was quantified using RSEM version 1.3.1 [70], and differential gene expression analyses were performed using EBSeq with FDR cutoff at 0.05 [71]. The alignment and quantification were performed individually for each replicate, and the quantified read counts for both replicates were provided as an input to EBSeq. Gene z-score values of the 284 most differentially expressed genes were used for heatmap and Venn diagrams [72] in Fig 2D. Sample z-scores of the log normalized read counts (log2[RPKM/FPKM] + 1) for the 284 most differentially expressed genes were used to compute Pearson correlation coefficients between different datasets.

### Data and software

Processed data underlying graphs in all figures are provided in S1–S10 Data. For RNA-seq data, normalized read count values quantified as FPKM are provided in S1 Table. Sequencing data are also deposited in Gene Expression Omnibus (GEO) repository with accession number GSE137492. MATLAB scripts for analyzing experimental data can be obtained from https://github.com/sc65/CellTracker and for running simulations from https://github.com/sc65/pde_simulation. Simulations for the activator-inhibitor model in Fig 3 can be reproduced by running the code runFile_fft.m in the Github repository sc65/pde_simulation/newFunctions.

## Supporting information

S1 Fig Fate patterning requires WNT and NODAL signaling, related to Fig 1. (A) Images of samples immunostained for SOX17 in different conditions—control and *NODAL* −/− cells were treated with 50 ng/ml BMP4. WNT inhibition indicates treatment of wild-type cells with 50 ng/ml BMP and 5 μM IWP2. All samples were fixed 44 h post treatment. Quantification represents intensity levels of indicated markers normalized to DAPI, averaged at different positions along the colony radii (radial averages, S9 Fig). Error bars represent standard error of the mean. *N* ≥ 10. Colony diameter = 700 μm. (B) Schematic showing NODAL, TGF-b binding to receptors and nuclear translocation of signal transducer SMAD2. TGF-beta inhibitor SB blocks the activity of TGF-beta type 1 receptors ALK4, 5, and 7 and thereby inhibits downstream signaling. NODAL knockout cells are incapable of NODAL production. (C) Images of samples immunostained for SMAD2 after 44 h of BMP4 treatment in different conditions as indicated above colonies and with concentrations as in (D). Squares indicate position of high-magnification small images adjacent to each condition. Scale bar = 100 μm. (D, E) Images of samples immunostained for indicated markers after 44 h of treatment with 50 ng/ml BMP4 and 10 μM SB (BMP4 + SB). Quantification represents intensity levels of indicated markers normalized to DAPI, averaged at different positions along the colony radii in the SB-treated, control, and NODAL knockout samples. N ≥ 10.
(TIF)

S2 Fig Creation and validation of NODAL knockout cells, related to Fig 1. (A) sgRNA used to make a double-stranded break on exon1 of endogenous *NODAL* gene. (B) Images of NODAL knockout cells immunostained for pluripotency markers OCT4, NANOG, SOX2 at passage 34 and passage 50. Histograms represent marker levels normalized to DAPI. *N* > 1,000 cells. (C) Western blot for NODAL following treatment with 10 μM CHIR in wild-type ESI017 cells and NODAL knockout cells. (D) Genomic sequence of *NODAL* locus in NODAL knockout cells.
(TIF)

S3 Fig Edge cells of BMP-treated micropatterned hESCs recapitulate cell fate of BMP-treated hESCs in regular culture, related to Fig 2. (A) Images of samples immunostained for the indicated markers at 48 h post BMP treatment in different conditions. No BMP was added in mTeSR sample. Quantification represents average mean intensity levels per cell of indicated markers normalized to DAPI. *N*cells > 500. Error bars represent standard deviation across cells. (B) Images of samples immunostained for the indicated markers at 44 h post BMP treatment in different conditions—BMP4 only, BMP and IWP2, and BMP4 and SB. Quantification represents intensity levels of indicated markers normalized to DAPI, averaged at different positions along the colony radii. Error bars represent standard error of the mean. *N* ≥ 10. Scale bar = 100 μm. (C) Histogram showing log values of absolute fold change of differentially expressed genes between different samples. (D) Pearson correlation coefficients for lineage-specific genes in the human embryo dataset. (E) Raw read counts for indicated genes in different samples.
(TIF)

S4 Fig WNT signaling dynamics lie outside the Turing instability regime, related to Fig 3. (A) Equations and simulations for stripe-forming Turing patterns. Simulation domain, assumptions, and initial conditions are the same as defined in Fig 3. D_A_ = 0.005, D_I_ = 0.2, s_A_ = 0.1, s_I_ = 0.2, kd_A_ = 0.1, kd_I_ = 0.2, κ_A_ = 0.25. degradation rate outside colony (kd = 0.5). (B) Average nonmembrane beta-catenin levels as a function of radial position at different times post BMP treatment. (C) Threshold signaling (dotted line) defined as the half-maximum of average nonmembrane beta-catenin levels at time point when signaling peak is the highest (38 h). *n* = 9. Error bars indicate standard error.
(TIF)

S5 Fig Cell division and cell movement during fate patterning, related to Fig 4. (A) (Top) Snapshots from time-lapse imaging of well-mixed populations of different cell populations at indicated times. Negative control: ESI017-CFP-H2B cells, ESI017-RFP-H2B cells. Positive control: ESI017-CFP-H2B cells, ESI017-RFP-H2B cells predifferentiated to extra-embryonic CDX2+ fate. Experimental condition: ESI017-CFP-H2B cells, RUES-VENUS-H2B cells. (Bottom) Quantification represents fraction of cells with more than 60% similar-cell (same cell type) neighbors (similarity index). A cell within a distance of 62 μm is defined as a neighbor. *N* > 400. (B) Number of progeny of tracked cells that start in the outer, inner, or center regions as defined in Fig 4. No significant difference between cell division trends across 3 regions. MATLAB function kstest2 returned 0 for all three comparisons. 0 progeny: No cell division, 2 progeny: 1 cell division, 3 progeny: 1 daughter cell divides, 4 progeny: both daughter cells divide (pictorial representation adjacent to figure). (C) Histogram of cell cycle time of daughter cells that divided during imaging (time to go from red cells to orange cells in pictorial representation of progeny number). (D) Histogram of distance moved by cells. (E) Histogram of radial displacement. (F) Histogram of angular displacement. Distance moved along the arc is considered as a proxy for angular displacement. (G) Distance moved by cells as a function of their displacement. (H) Angular displacement as a function of radial displacement.
(TIF)

S6 Fig WNT signaling dynamics in LDN- and IWP2-treated samples related to Fig 5. (A) Images of colonies immunostained for pSMAD1 and DAPI after 44 h of BMP treatment. The time between BMP4 and LDN addition is indicated above the image. No LDN was added in the control sample. Quantification represents average nuclear intensities of indicated markers normalized to DAPI as a function of radial position. *N* ≥ 5. (B, F) Average nonmembrane β-catenin levels as a function of radial position. The time in the legend represents time post BMP treatment being analyzed in each curve. The time above the curves indicate the time between BMP4 and LDN/IWP2 treatment. No LDN or IWP2 was added in control. (C, F) Average nonmembrane β-catenin levels at the time point when signaling is highest in the control sample. This time point was used to define threshold signaling levels (dashed line) for each condition. (D) SMAD2 immunostaining 44 h post BMP treatment under indicated conditions. Control represents BMP-treated wild-type cells. (E) SMAD2 immunostaining 44 h post BMP treatment. IWP2 was added at 25 h post BMP treatment. For all radial averages plot, error bars represent standard error. Scale bar = 100 μm.
(TIF)

S7 Fig A single threshold in WNT signaling is insufficient to determine mesoderm differentiation, related to Fig 9. (A) GFP-β-catenin hESCs immunostained for BRA and DAPI following 47 h time-lapse imaging. Scale bar: 100 μm. (B) (Left) Average BRA intensity levels and nonmembrane β-catenin at highest signaling as a function of radial position. (Right) Average BRA intensity levels as a function of nonmembrane β-catenin color-coded by edge distance (μm). (C) Average BRA intensity levels, nonmembrane β-catenin at highest signaling (red curve) and last time point (47 h, gray curve) as a function of radial position for individual colonies. In Colony 8, highest signaling occurs at the last time point. (D) GFP-β-catenin hESCs immunostained for BRA 47 h post treatment with BMP4. LDN was added at 13 h post BMP treatment in the LDN at 13 h sample. (Right) Quantified average nonmembrane β-catenin and BRA levels as a function of edge distance in the 2 conditions. *N* = 8. Error bar represent standard error across colonies.
(TIF)

S8 Fig Computing fate territories from experimental data, related to Fig 10. (A) Simulated time evolution of WNT and NODAL (B) Map of mean DAPI intensity as a proxy for cell density in experiments on different shapes. Each map is normalized so that the maximum nuclear intensity in circular colonies at 44 h post BMP treatment is 1. (C) Histograms of DAPI intensities in 3 different shapes; all three had similar means in the normalized units defined above (circle: 0.71, triangle: 0.69, pacman: 0.63) indicating comparable cell-seeding densities. (D) Intensity maps of indicated fate markers, normalized to the maximum intensity for the same marker in circular colonies. Fate territories were assigned by selecting the fate marker with the maximum intensity in that region. The nuclear and fate intensity maps represent values averaged over *N* = 18 colonies in each of the 3 shapes.
(TIF)

S9 Fig Analyses supplement. All quantifications of micropatterned data, plotted as intensity versus radial position (distance from colony edge) in the figures were created using one of the following two methods. (I) Normalized nuclear intensity: (1) For each colony, a nuclear mask was created by segmenting the DAPI image of that colony in Ilastik ([66]; panel A). (2) The nuclear mask was then applied to each channel to extract nuclear pixels in that channel (as shown in panel B in the image labelled as Channel1_nuclear). (3) For each nuclear pixel in the image Channel1_nuclear, average local intensity was calculated in a region of radius 120 μm (as shown in panel B in the image labelled as Channel1_nuclear_average). (4) Steps 2 and 3 were applied to DAPI image to get DAPI_Average (panel C). (5) Channel1_nuclear_average was normalized by DAPI_Average to get Channel1_nuclearToDAPI (panel D). (6) Mean intensity was calculated in different bins along the radius of Channel1_nuclearToDAPI to get radial averages. (7) Mean intensity in each bin was averaged across all colonies to get average radial average intensities (panel E). The final intensities were normalized to the intensity values in the same channel in the control sample (treated with BMP4 for 44 h). Datasets quantified: fate data and SMAD data (Figs 1, 2A, 2H, 4C and 6–9). (II) Non-membrane β-catenin intensities. (1) For each colony, a membrane mask and a colony mask were created in Ilastik [66] using the GFP-β-catenin image (panels A, B). (2) Membrane mask was subtracted from colony mask to get nonmembrane mask (panel B). (3) Nonmembrane mask was applied to GFP-β-catenin image to extract nonmembrane β-catenin pixels (panel C). 4) As in (I), average intensity was calculated in different bins along the radius of Colony1_non-membrane images across different colonies. Datasets quantified: all GFP-β-catenin movies (Figs 3C, 5 and 9C). Error bars indicate standard error (panel D). Scale bar: 100 μm.
(TIF)

S1 Movie Simulation of activator-inhibitor model in a circular colony, within spot forming Turing regime, related to Fig 3B. (MOV)

S2 Movie Simulation of activator-inhibitor model in a lattice, within spot forming Turing regime, related to Fig 3B. (MOV)

S3 Movie Simulation of activator-inhibitor model in a circular colony, outside Turing regime, related to Fig 3B. (MOV)

S4 Movie Simulation of activator-inhibitor model in a lattice, outside Turing regime, related to Fig 3B. (MOV)

S5 Movie Simulation of activator-inhibitor model in a circular colony, within stripe forming Turing regime, related to Fig 3A and 3B. (MOV)

S6 Movie Simulation of activator-inhibitor model in a lattice, within stripe forming Turing regime, related to Fig 3A and 3B. (MOV)

S7 Movie WNT signaling dynamics during fate patterning, related to Fig 3C. GFP-β-catenin hESCs treated with 50 ng/ml BMP4, imaged from 3–47 h post treatment. Only nonmembrane regions are shown. Colony diameter: 800 um.
(MOV)

S8 Movie Cell movement during patterning, related to Fig 6A. Cells with a nuclear fluorescent marker (RUES2-VENUS-H2B, green cells) mixed with unlabeled cells (ESI017) in the ratio 1:100, imaged from 20 to 47.5 h post BMP treatment.
(AVI)

S9 Movie WNT signaling dynamics with LDN addition at 0 h, related to Fig 5A. GFP-β-catenin hESCs treated with 200 nM LDN at 0 h and 50 ng/ml BMP4, imaged from 2–46 h post treatment. Only nonmembrane regions are shown. Colony size: 700 um.
(AVI)

S10 Movie WNT signaling dynamics with LDN addition at 11 h, related to Fig 5A. GFP-β-catenin hESCs treated with 50 ng/ml BMP4, imaged from 2–46 h post treatment; 200 nM LDN was added 11 h post BMP treatment. Only nonmembrane regions are shown. Colony size: 700 um.
(AVI)

S11 Movie WNT signaling dynamics with LDN addition at 23 h, related to Fig 5A. GFP-β-catenin hESCs treated with 50 ng/ml BMP4, imaged from 2–46 h post treatment; 200 nM LDN was added 23 h post BMP treatment. Only nonmembrane regions are shown. Colony size: 700 um.
(AVI)

S12 Movie WNT signaling dynamics with No LDN addition, related to Fig 5A. GFP-β-catenin hESCs treated with 50 ng/ml BMP4, imaged from 2–46 h post treatment. No LDN was added in this sample. Only nonmembrane regions are shown. Colony size: 700 um.
(AVI)

S13 Movie WNT signaling dynamics with IWP2 addition at 15 h, related to Fig 5E. GFP-β-catenin hESCs treated with 50 ng/ml BMP4 and 5 μM IWP2, imaged from 2–47 h post treatment. Only nonmembrane regions are shown. Colony size: 700 μm.
(AVI)

S14 Movie WNT signaling dynamics with IWP2 addition at 30 h, related to Fig 5E. GFP-β-catenin hESCs treated with 50 ng/ml BMP4, imaged from 2–47 h post treatment. Only nonmembrane regions are shown. Colony size: 700 μm.
(AVI)

S15 Movie WNT signaling dynamics with no IWP2 addition, related to Fig 5E. GFP-β-catenin hESCs treated with 50 ng/ml BMP4, imaged from 2–46 h post treatment. No IWP2 was added in this sample. Only nonmembrane regions are shown. Colony size: 700 μm.
(AVI)

S16 Movie BMP dynamics used as an input for WNT and NODAL, related to Fig 10A. (MOV)

S17 Movie WNT dynamics, related to Fig 10A. (MOV)

S18 Movie NODAL dynamics, related to Fig 10A. (MOV)

S1 Table Normalized gene expression values and gene lists, related to Fig 2. (Sheet1) Fragments per kilobase per million (FPKM) values for all genes in all hESC samples. Sample_1, Sample_2, Sample_avg represent 2 biological replicates and average of the 2, respectively. (Sheet2) The list of 284 differentially expressed genes in BMP-treated samples versus pluripotent samples. (Sheet 3) The list of 174 lineage-specific genes in the human embryo dataset.
(XLSX)

S1 Model Activator-inhibitor model to test the role of Turing instability in signaling dynamics. (PDF)

S2 Model WNT and NODAL reaction-diffusion model. (PDF)

S1 Data Radial intensity profiles for indicated markers for individual colonies. (MAT)

S2 Data Intensity profiles and differentially expressed genes list. (MAT)

S3 Data β-catenin intensity profiles for individual colonies at all imaging time points. (MAT)

S4 Data Cell movement statistics. (MAT)

S5 Data β-catenin intensity profiles for individual colonies at all imaging time points under specified conditions. (MAT)

S6 Data SMAD2 intensity profiles for individual colonies under specified conditions. (MAT)

S7 Data pSMAD1 intensity profiles for individual colonies under specified conditions. (MAT)

S8 Data CDX2 intensity profiles for individual colonies under specified conditions. (MAT)

S9 Data BRA intensity profiles for individual colonies under specified conditions. (MAT)

S10 Data Simulated BMP, WNT, and NODAL signaling levels under specified conditions, related to S2 Model. (MAT)

S1 Text Explanation of variables in supporting information data files (S1–S10 Data). (PDF)

## Abbreviations

BMP: Bone Morphogenic Protein
BRA: BRACHYURY
CT: cytotrophoblast
E: embryonic day
FDR: false discovery rate
FPKM: fragments per kilobase of transcript per million mapped reads
GEO: Gene Expression Omnibus
GFP: Green Fluorescent Protein
hESC: human embryonic stem cell
LDN: LDN193189
MEF-CM: mouse embryonic fibroblast conditioned media
PE: primitive endoderm
PVDF: polyvinyl difluoride
RNA-seq: RNA sequencing
RPKM: Reads Per Kilobase of transcript per Million mapped reads
SB: SB431542
TGFβ: Transforming Growth Factor β1
