# Supplementary figures and images for: Dissecting the dynamics of signaling events in the BMP, WNT, and NODAL cascade during self-organized fate patterning in human gastruloids
Source: PLoS Biol. 2019 Oct 15;17(10):e3000498. doi: 10.1371/journal.pbio.3000498 (PMC6814242; doi:10.1371/journal.pbio.3000498)

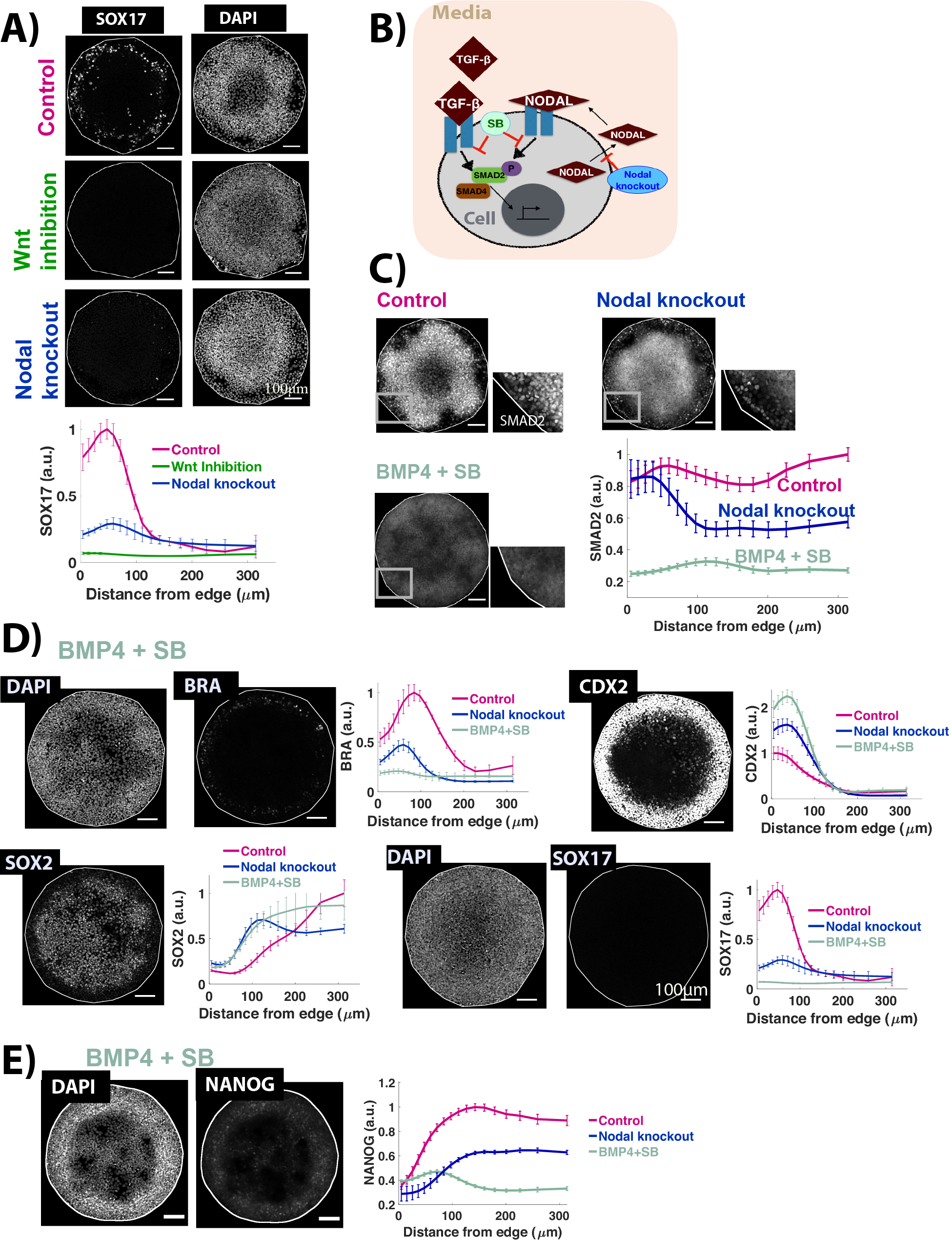

Supplement: S1 Fig — (A) Images of samples immunostained for SOX17 in different conditions—control and NODAL −/− cells were treated with 50 ng/ml BMP4. WNT inhibition indicates treatment of wild-type cells with 50 ng/ml BMP and 5 μM IWP2. All samples were fixed 44 h post treatment. Quantification represents intensity levels of indicated markers normalized to DAPI, averaged at different positions along the colony radii (radial averages, S9 Fig). Error bars represent standard error of the mean. N ≥ 10. Colony diameter = 700 μm. (B) Schematic showing NODAL, TGF-b binding to receptors and nuclear translocation of signal transducer SMAD2. TGF-beta inhibitor SB blocks the activity of TGF-beta type 1 receptors ALK4, 5, and 7 and thereby inhibits downstream signaling. NODAL knockout cells are incapable of NODAL production. (C) Images of samples immunostained for SMAD2 after 44 h of BMP4 treatment in different conditions as indicated above colonies and with concentrations as in (D). Squares indicate position of high-magnification small images adjacent to each condition. Scale bar = 100 μm. (D, E) Images of samples immunostained for indicated markers after 44 h of treatment with 50 ng/ml BMP4 and 10 μM SB (BMP4 + SB). Quantification represents intensity levels of indicated markers normalized to DAPI, averaged at different positions along the colony radii in the SB-treated, control, and NODAL knockout samples. N ≥ 10. (TIF) [file pbio.3000498.s001.tif]

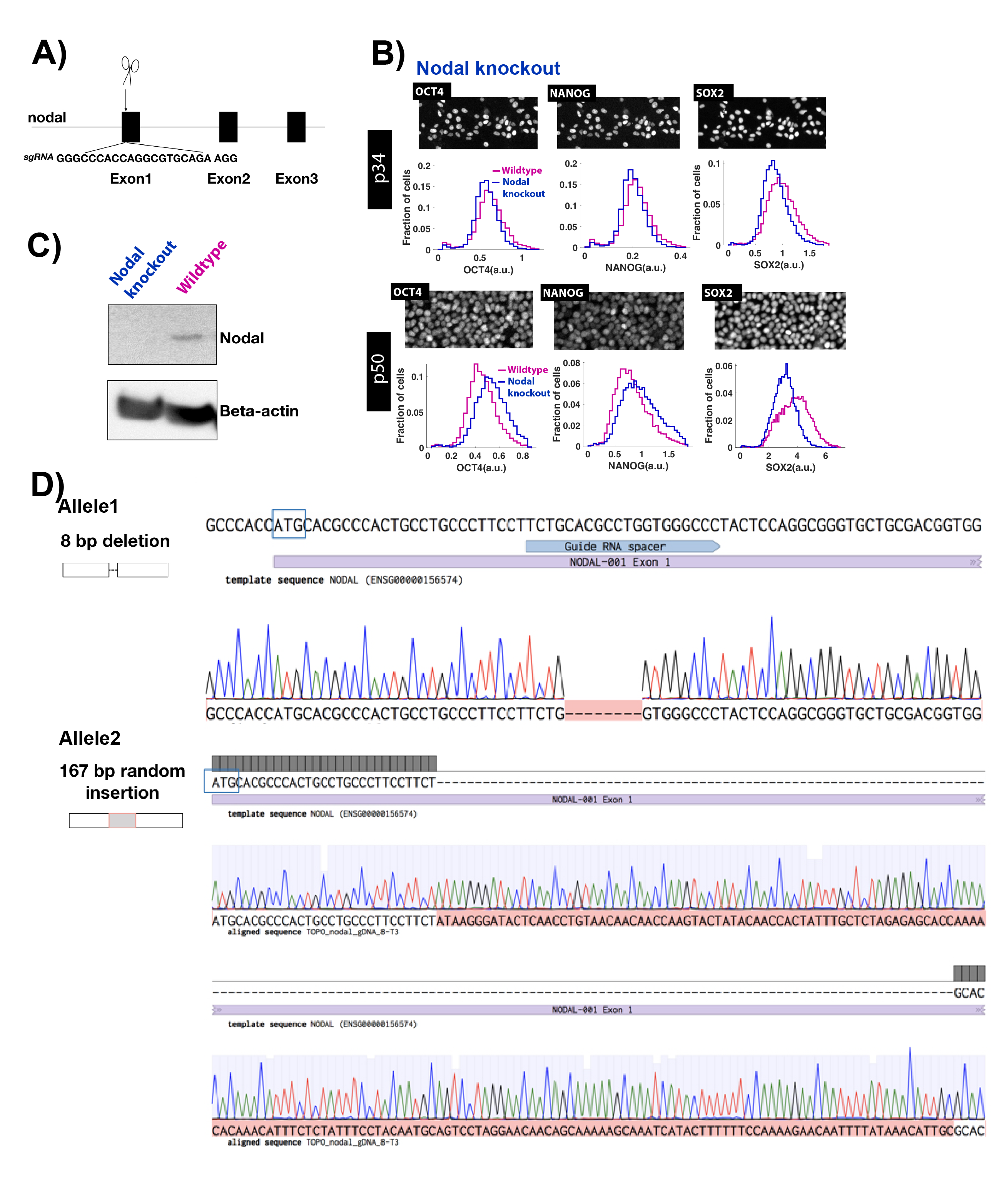

Supplement: S2 Fig — (A) sgRNA used to make a double-stranded break on exon1 of endogenous NODAL gene. (B) Images of NODAL knockout cells immunostained for pluripotency markers OCT4, NANOG, SOX2 at passage 34 and passage 50. Histograms represent marker levels normalized to DAPI. N > 1,000 cells. (C) Western blot for NODAL following treatment with 10 μM CHIR in wild-type ESI017 cells and NODAL knockout cells. (D) Genomic sequence of NODAL locus in NODAL knockout cells. (TIF) [file pbio.3000498.s002.tif]

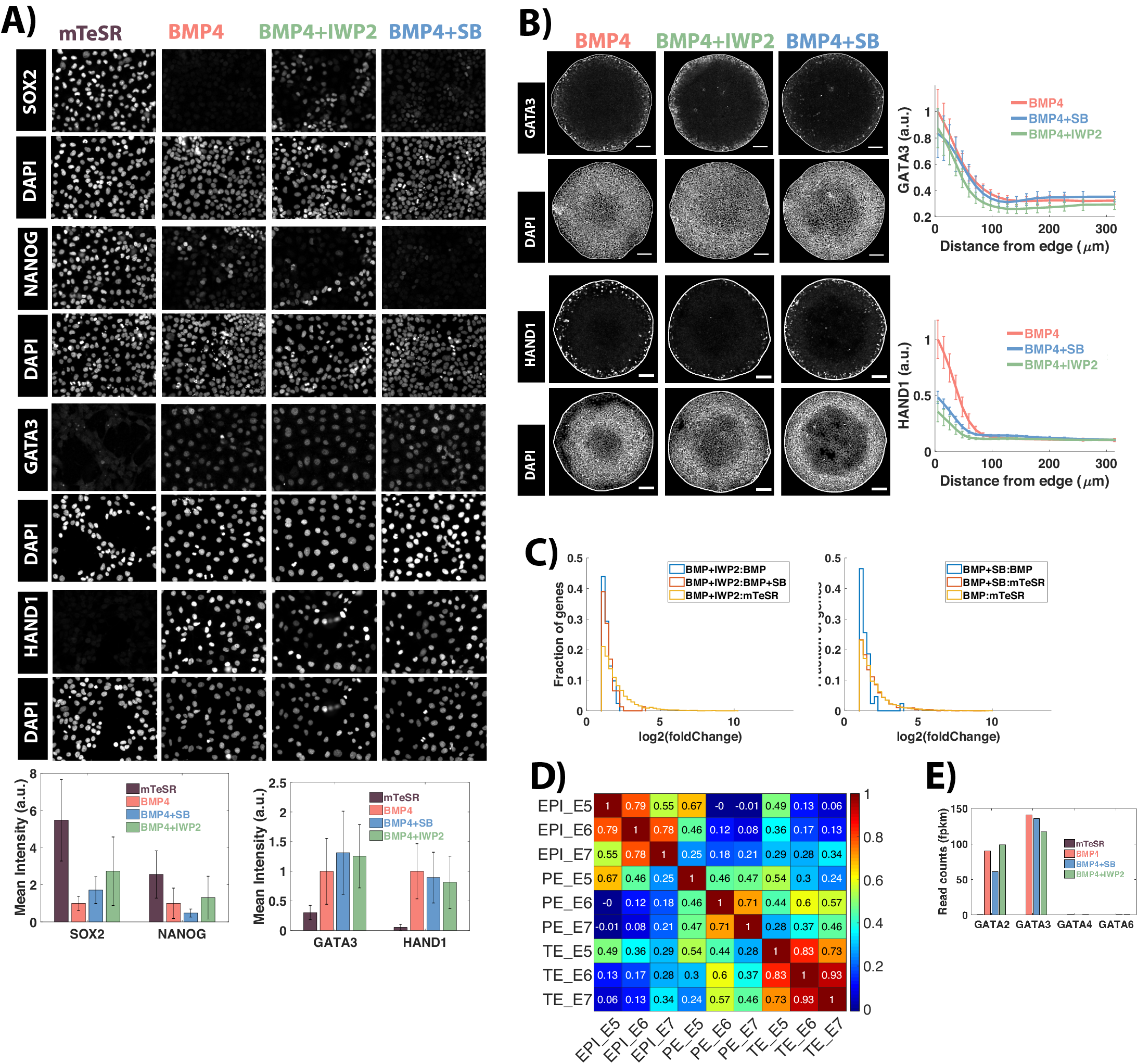

Supplement: S3 Fig — (A) Images of samples immunostained for the indicated markers at 48 h post BMP treatment in different conditions. No BMP was added in mTeSR sample. Quantification represents average mean intensity levels per cell of indicated markers normalized to DAPI. Ncells > 500. Error bars represent standard deviation across cells. (B) Images of samples immunostained for the indicated markers at 44 h post BMP treatment in different conditions—BMP4 only, BMP and IWP2, and BMP4 and SB. Quantification represents intensity levels of indicated markers normalized to DAPI, averaged at different positions along the colony radii. Error bars represent standard error of the mean. N ≥ 10. Scale bar = 100 μm. (C) Histogram showing log values of absolute fold change of differentially expressed genes between different samples. (D) Pearson correlation coefficients for lineage-specific genes in the human embryo dataset. (E) Raw read counts for indicated genes in different samples. (TIF) [file pbio.3000498.s003.tif]

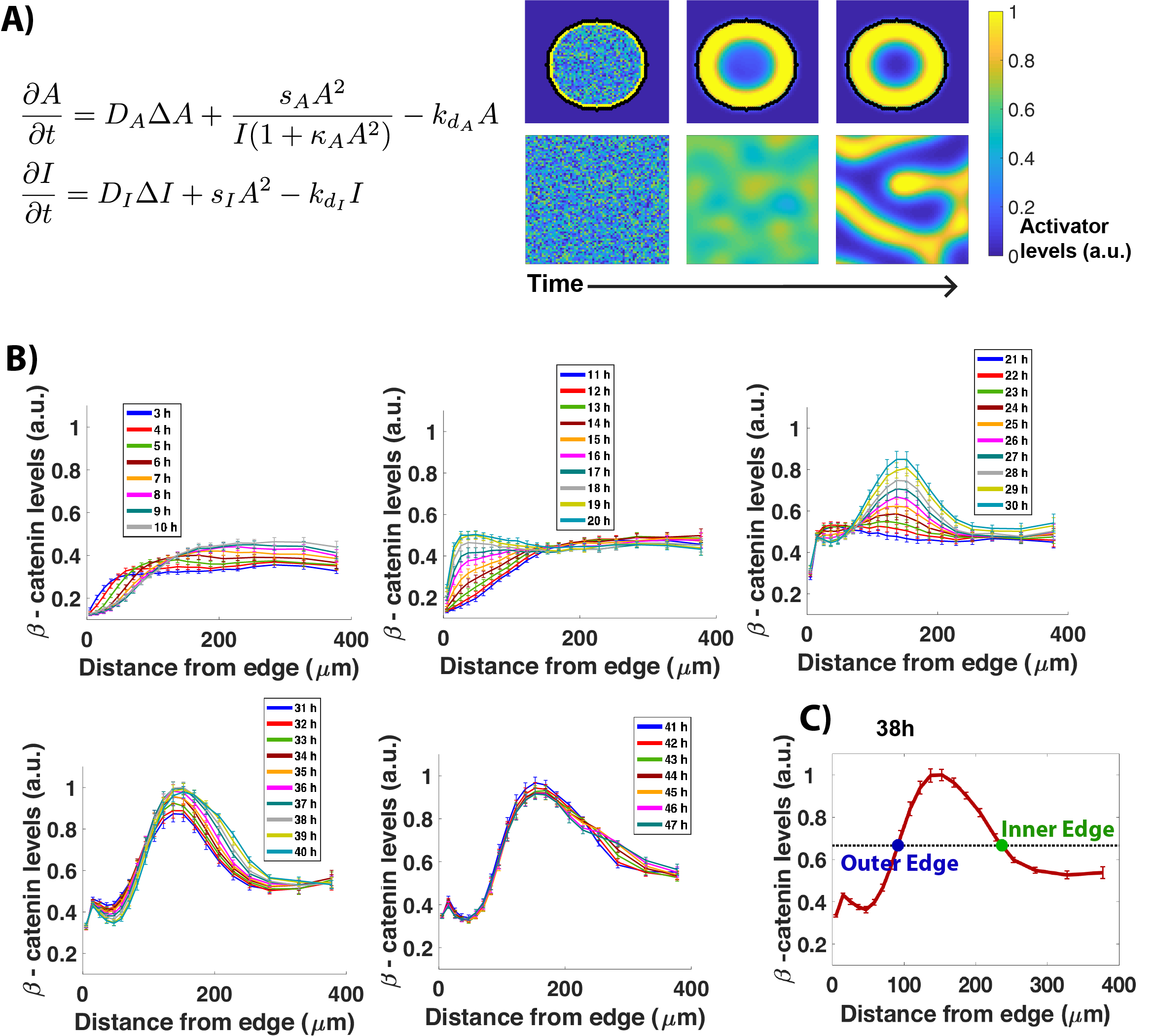

Supplement: S4 Fig — (A) Equations and simulations for stripe-forming Turing patterns. Simulation domain, assumptions, and initial conditions are the same as defined in Fig 3. DA = 0.005, DI = 0.2, sA = 0.1, sI = 0.2, kdA = 0.1, kdI = 0.2, κA = 0.25. degradation rate outside colony (kd = 0.5). (B) Average nonmembrane beta-catenin levels as a function of radial position at different times post BMP treatment. (C) Threshold signaling (dotted line) defined as the half-maximum of average nonmembrane beta-catenin levels at time point when signaling peak is the highest (38 h). n = 9. Error bars indicate standard error. (TIF) [file pbio.3000498.s004.tif]

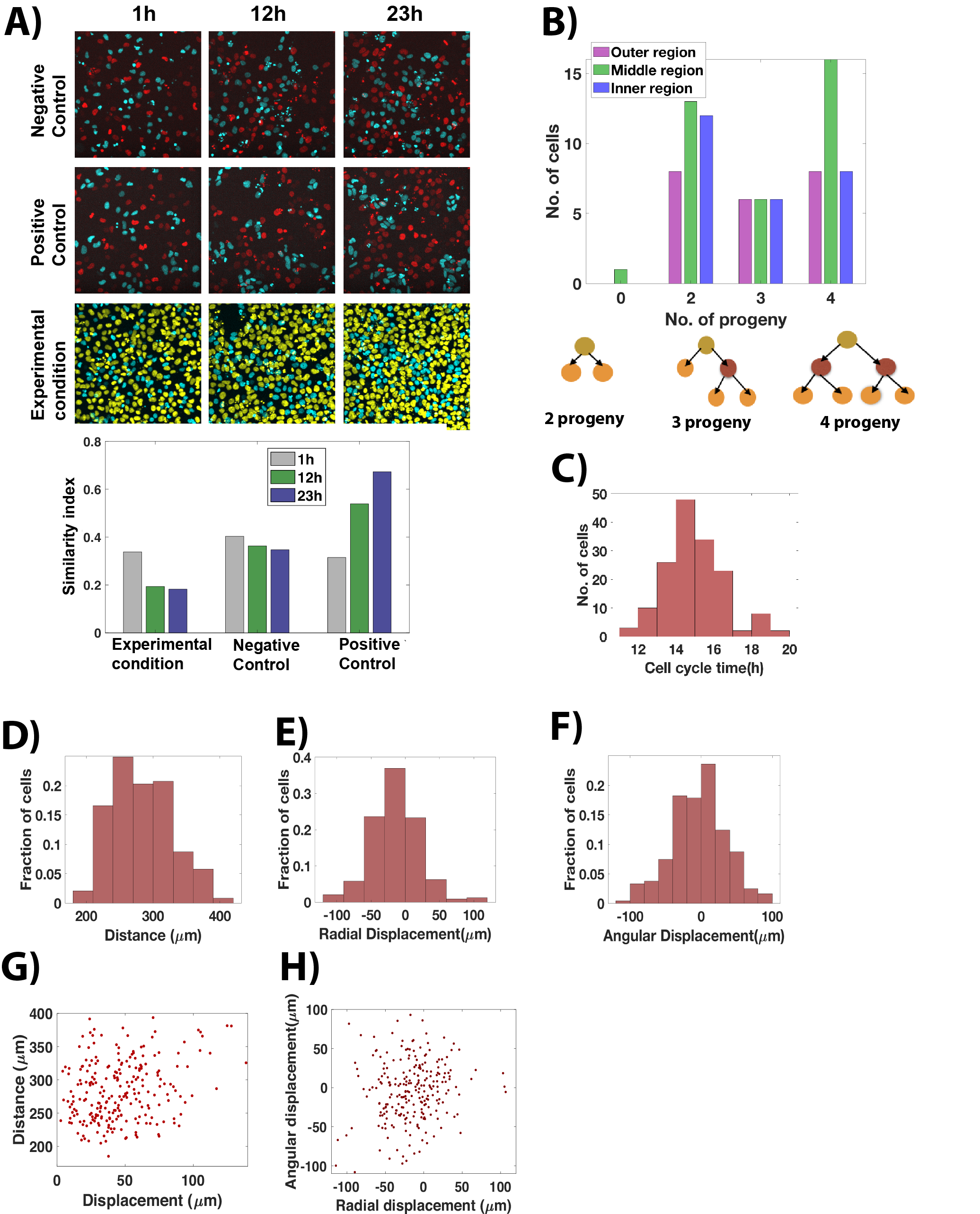

Supplement: S5 Fig — (A) (Top) Snapshots from time-lapse imaging of well-mixed populations of different cell populations at indicated times. Negative control: ESI017-CFP-H2B cells, ESI017-RFP-H2B cells. Positive control: ESI017-CFP-H2B cells, ESI017-RFP-H2B cells predifferentiated to extra-embryonic CDX2+ fate. Experimental condition: ESI017-CFP-H2B cells, RUES-VENUS-H2B cells. (Bottom) Quantification represents fraction of cells with more than 60% similar-cell (same cell type) neighbors (similarity index). A cell within a distance of 62 μm is defined as a neighbor. N > 400. (B) Number of progeny of tracked cells that start in the outer, inner, or center regions as defined in Fig 4. No significant difference between cell division trends across 3 regions. MATLAB function kstest2 returned 0 for all three comparisons. 0 progeny: No cell division, 2 progeny: 1 cell division, 3 progeny: 1 daughter cell divides, 4 progeny: both daughter cells divide (pictorial representation adjacent to figure). (C) Histogram of cell cycle time of daughter cells that divided during imaging (time to go from red cells to orange cells in pictorial representation of progeny number). (D) Histogram of distance moved by cells. (E) Histogram of radial displacement. (F) Histogram of angular displacement. Distance moved along the arc is considered as a proxy for angular displacement. (G) Distance moved by cells as a function of their displacement. (H) Angular displacement as a function of radial displacement. (TIF) [file pbio.3000498.s005.tif]

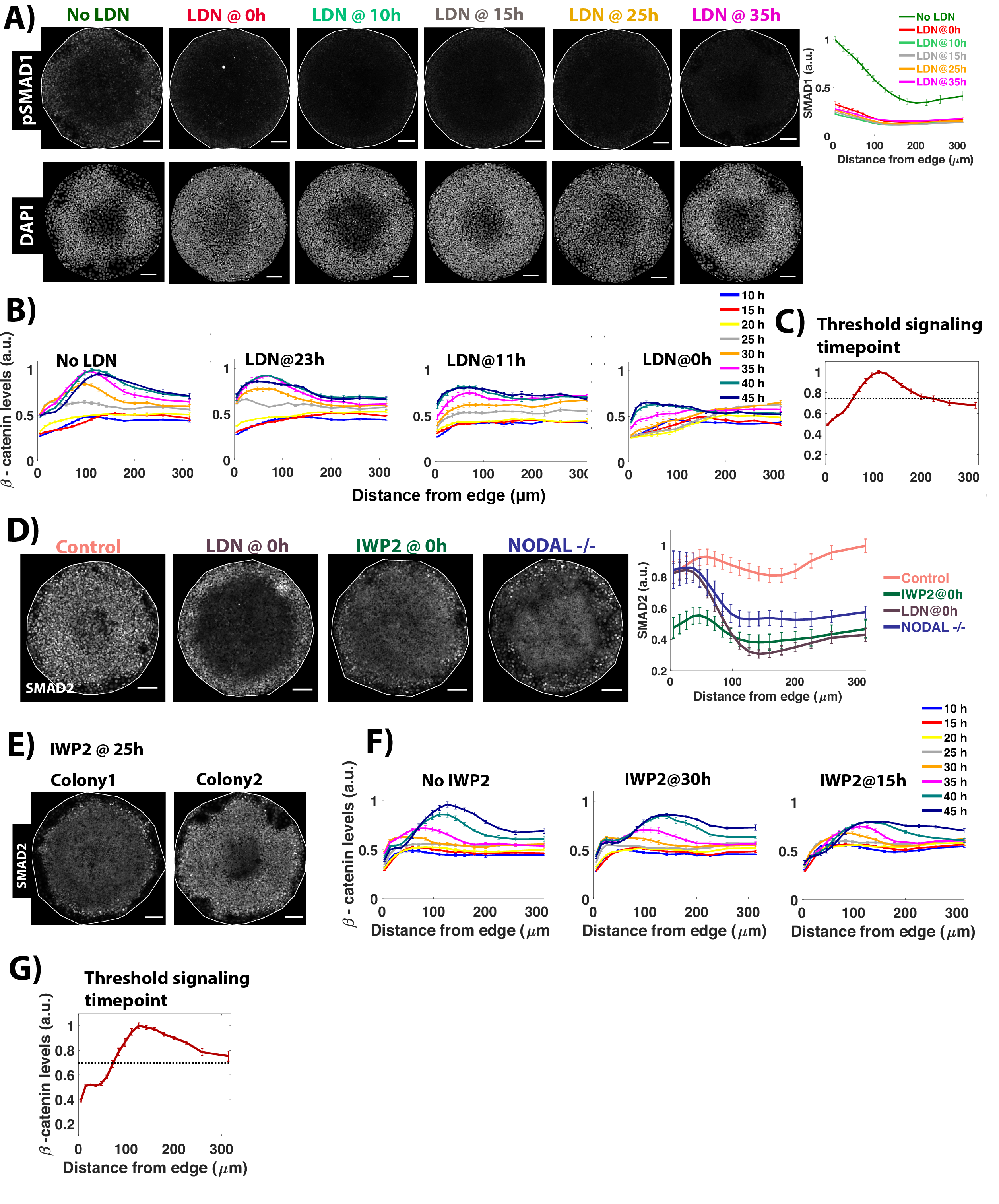

Supplement: S6 Fig — (A) Images of colonies immunostained for pSMAD1 and DAPI after 44 h of BMP treatment. The time between BMP4 and LDN addition is indicated above the image. No LDN was added in the control sample. Quantification represents average nuclear intensities of indicated markers normalized to DAPI as a function of radial position. N ≥ 5. (B, F) Average nonmembrane β-catenin levels as a function of radial position. The time in the legend represents time post BMP treatment being analyzed in each curve. The time above the curves indicate the time between BMP4 and LDN/IWP2 treatment. No LDN or IWP2 was added in control. (C, F) Average nonmembrane β-catenin levels at the time point when signaling is highest in the control sample. This time point was used to define threshold signaling levels (dashed line) for each condition. (D) SMAD2 immunostaining 44 h post BMP treatment under indicated conditions. Control represents BMP-treated wild-type cells. (E) SMAD2 immunostaining 44 h post BMP treatment. IWP2 was added at 25 h post BMP treatment. For all radial averages plot, error bars represent standard error. Scale bar = 100 μm. (TIF) [file pbio.3000498.s006.tif]

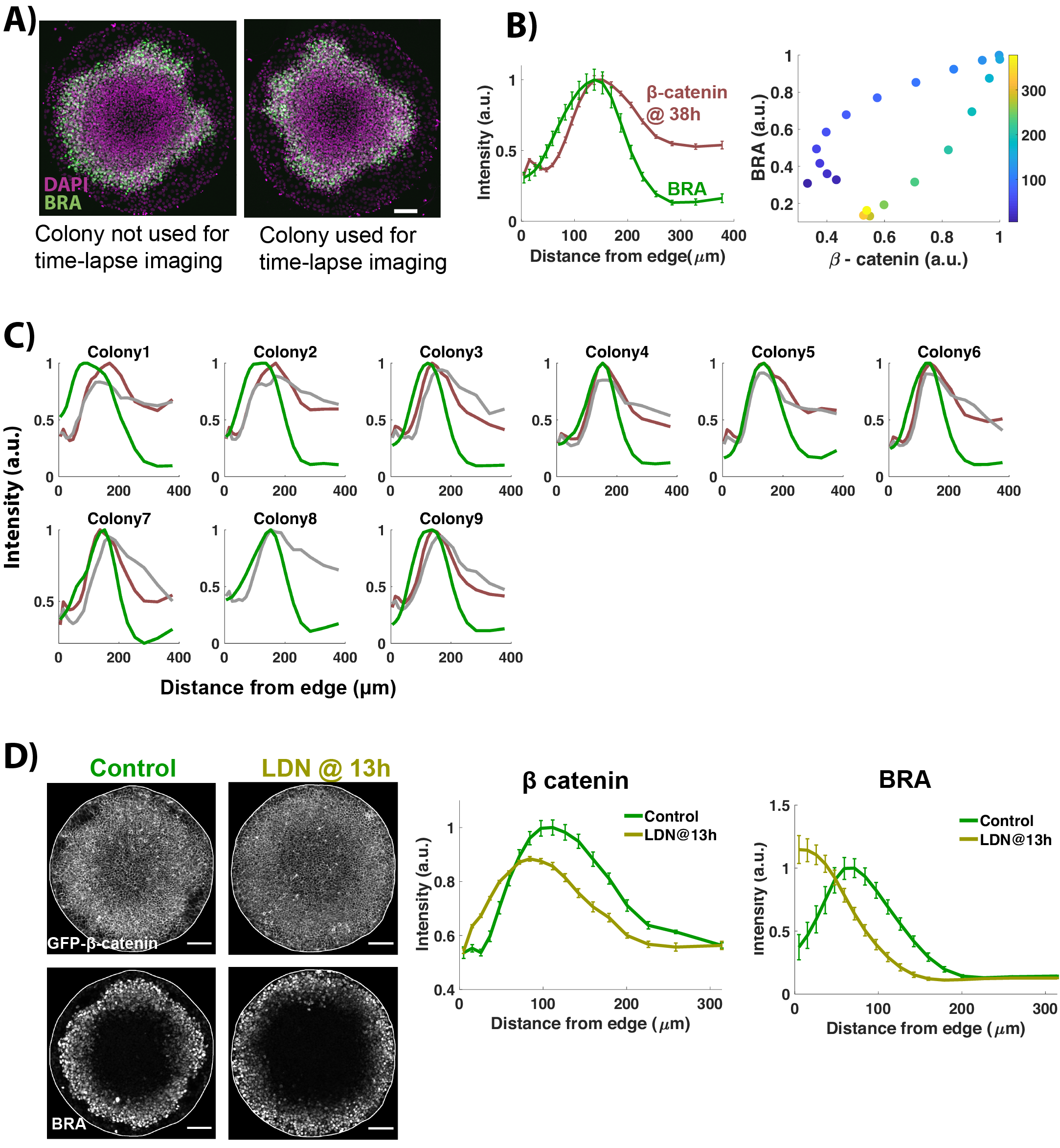

Supplement: S7 Fig — (A) GFP-β-catenin hESCs immunostained for BRA and DAPI following 47 h time-lapse imaging. Scale bar: 100 μm. (B) (Left) Average BRA intensity levels and nonmembrane β-catenin at highest signaling as a function of radial position. (Right) Average BRA intensity levels as a function of nonmembrane β-catenin color-coded by edge distance (μm). (C) Average BRA intensity levels, nonmembrane β-catenin at highest signaling (red curve) and last time point (47 h, gray curve) as a function of radial position for individual colonies. In Colony 8, highest signaling occurs at the last time point. (D) GFP-β-catenin hESCs immunostained for BRA 47 h post treatment with BMP4. LDN was added at 13 h post BMP treatment in the LDN at 13 h sample. (Right) Quantified average nonmembrane β-catenin and BRA levels as a function of edge distance in the 2 conditions. N = 8. Error bar represent standard error across colonies. (TIF) [file pbio.3000498.s007.tif]

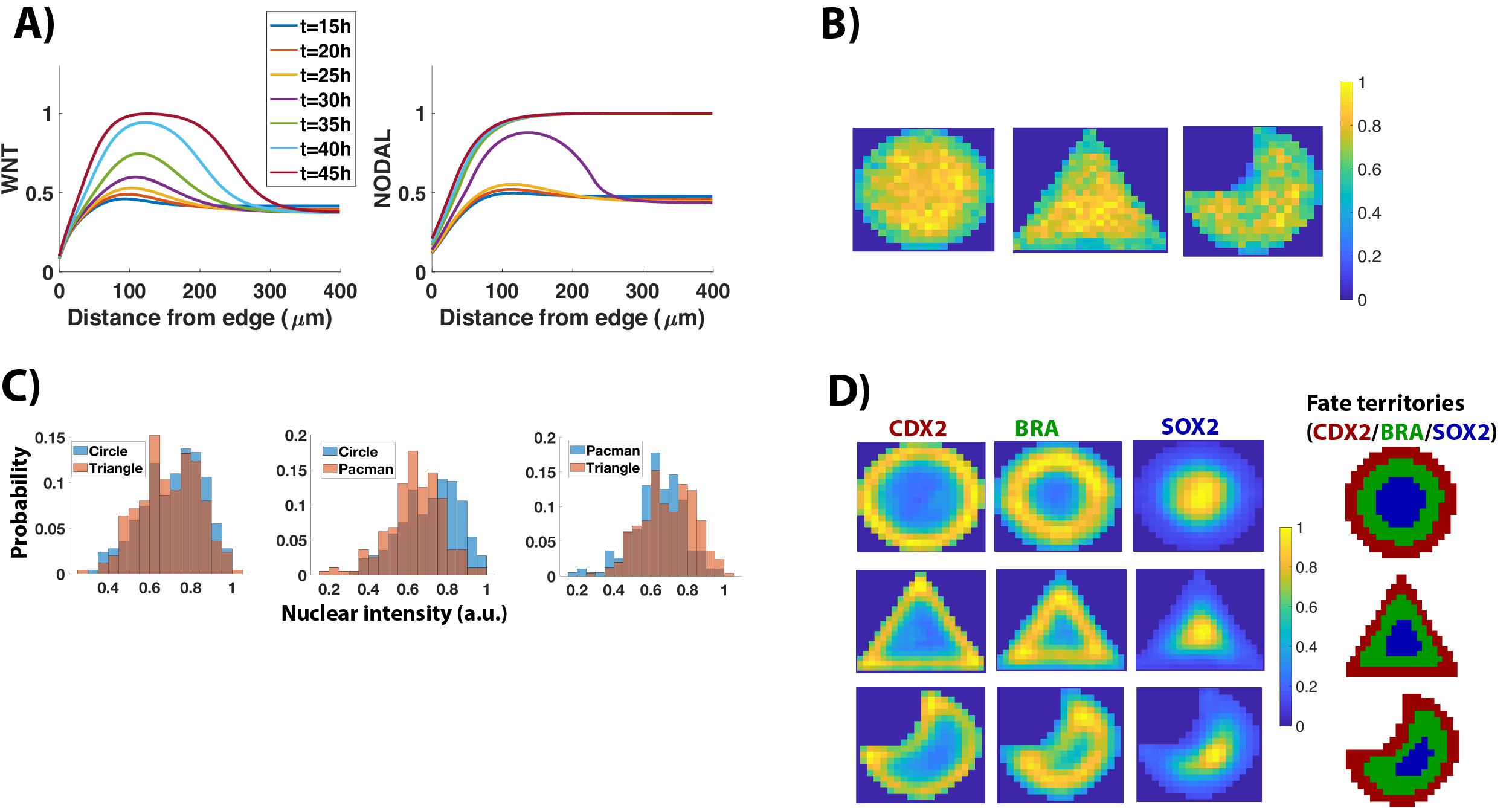

Supplement: S8 Fig — (A) Simulated time evolution of WNT and NODAL (B) Map of mean DAPI intensity as a proxy for cell density in experiments on different shapes. Each map is normalized so that the maximum nuclear intensity in circular colonies at 44 h post BMP treatment is 1. (C) Histograms of DAPI intensities in 3 different shapes; all three had similar means in the normalized units defined above (circle: 0.71, triangle: 0.69, pacman: 0.63) indicating comparable cell-seeding densities. (D) Intensity maps of indicated fate markers, normalized to the maximum intensity for the same marker in circular colonies. Fate territories were assigned by selecting the fate marker with the maximum intensity in that region. The nuclear and fate intensity maps represent values averaged over N = 18 colonies in each of the 3 shapes. (TIF) [file pbio.3000498.s008.tif]

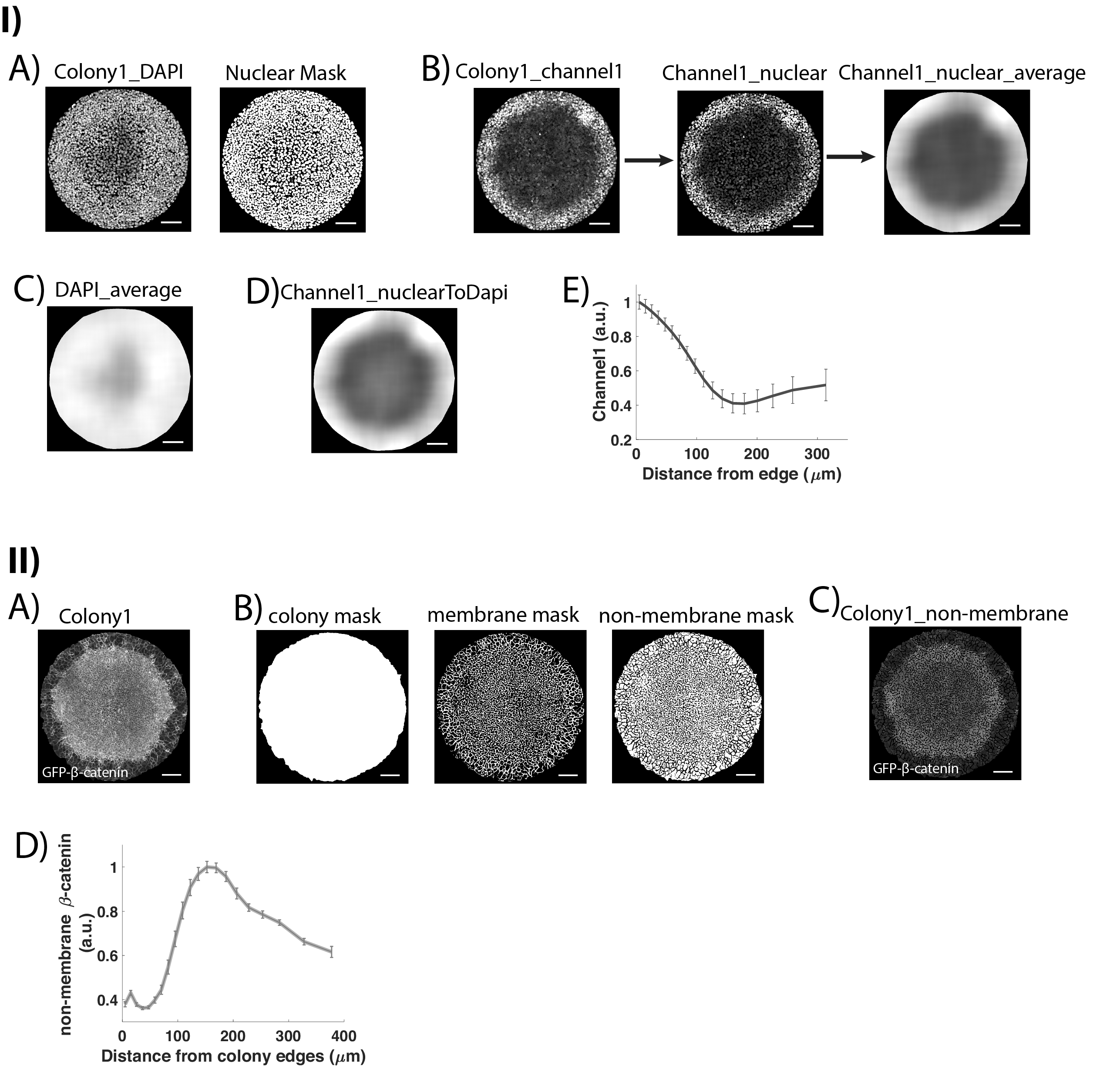

Supplement: S9 Fig — All quantifications of micropatterned data, plotted as intensity versus radial position (distance from colony edge) in the figures were created using one of the following two methods. (I) Normalized nuclear intensity: (1) For each colony, a nuclear mask was created by segmenting the DAPI image of that colony in Ilastik ([66]; panel A). (2) The nuclear mask was then applied to each channel to extract nuclear pixels in that channel (as shown in panel B in the image labelled as Channel1_nuclear). (3) For each nuclear pixel in the image Channel1_nuclear, average local intensity was calculated in a region of radius 120 μm (as shown in panel B in the image labelled as Channel1_nuclear_average). (4) Steps 2 and 3 were applied to DAPI image to get DAPI_Average (panel C). (5) Channel1_nuclear_average was normalized by DAPI_Average to get Channel1_nuclearToDAPI (panel D). (6) Mean intensity was calculated in different bins along the radius of Channel1_nuclearToDAPI to get radial averages. (7) Mean intensity in each bin was averaged across all colonies to get average radial average intensities (panel E). The final intensities were normalized to the intensity values in the same channel in the control sample (treated with BMP4 for 44 h). Datasets quantified: fate data and SMAD data (Figs 1, 2A, 2H, 4C and 6–9). (II) Non-membrane β-catenin intensities. (1) For each colony, a membrane mask and a colony mask were created in Ilastik [66] using the GFP-β-catenin image (panels A, B). (2) Membrane mask was subtracted from colony mask to get nonmembrane mask (panel B). (3) Nonmembrane mask was applied to GFP-β-catenin image to extract nonmembrane β-catenin pixels (panel C). 4) As in (I), average intensity was calculated in different bins along the radius of Colony1_non-membrane images across different colonies. Datasets quantified: all GFP-β-catenin movies (Figs 3C, 5 and 9C). Error bars indicate standard error (panel D). Scale bar: 100 μm. (TIF) [file pbio.3000498.s009.tif]
