# Supplementary material for: Dissecting the dynamics of signaling events in the BMP, WNT, and NODAL cascade during self-organized fate patterning in human gastruloids
Source: PLoS Biol. 2019 Oct 15;17(10):e3000498. doi: 10.1371/journal.pbio.3000498 (PMC6814242; doi:10.1371/journal.pbio.3000498)
Supplement: S1 Model — (PDF) [file pbio.3000498.s029.pdf]

Activator-Inhibitor Model

September 17, 2019

1 Introduction

Our experimental results show that paracrine Wnt and Nodal signals move through spatially confined human embryonic stem cell colonies treated with BMP4 (gastruloids) to initiate self-organized fate patterning. The movement of these signals can be recapitulated by a simple reaction-diffusion based activator-inhibitor model defined below, where the activator(A)-inhibitor(I) pair corresponds to a composite of Wnt/Nodal and their secreted, diffusible feedback inhibitors.

$$\frac{\partial A}{\partial t} = D_A \Delta A + \frac{s_A A^2}{(k_I + I)} - k_{d_A} A \tag{1.1}$$

$$\frac{\partial I}{\partial t} = D_I \Delta I + s_I A^2 - k_{d_I} I \tag{1.2}$$

The equations are adapted from equations 1 in [2]. In sections2 and 3, we follow the stability analyses given in [2] to show that the parameter regime that recapitulates inward movement of signals takes the system to a homogeneous steady state and not a Turing pattern.

2 Stability conditions

The qualitative behavior of the system of equations (1-2) depends on the choice of parameters. Although the possible parameter combinations are infinite, stability analyses can be used to determine parameter regimes corresponding to distinct system behaviors. Below, we derive conditions for the stability of the homogeneous steady state of the system and the formation spatial patterns.

To simplify analyses, we set $k_I + I = I'$. This transforms the equations to:

$$\frac{\partial A}{\partial t} = D_A \Delta A + \frac{s_A A^2}{I'} - k_{d_A} A \tag{2.1}$$

$$\frac{\partial I'}{\partial t} = D_I \Delta I' + s_I A^2 - k_{d_I}(I') + \sigma_I \tag{2.2}$$

where $\sigma_I = k_{d_I} * k_I$.

For simplicity, we assume that the system of equations operates in one dimensional closed domain [0,L], although the same analyses can be applied to a two-dimensional system. To non-dimensionalize the equations, we introduce new variables for length ($\bar{l}$), time ($\bar{t}$), and the concentrations ($\bar{A}$,$\bar{I}$) defined as:

$$\bar{t} = k_{d_A} * t$$
$$\bar{l} = (\sqrt{k_{d_A}/D_I}) * l$$
$$\bar{A} = \frac{k_{d_A} * s_I}{k_{D_I} * s_A} * A$$
$$\bar{I} = \frac{k_{d_A}^2 * s_I}{k_{D_I} * s_A^2} * I'$$

Rewriting equations (3),(4) with new variables yields:

$$\frac{\partial \bar{A}}{\partial \bar{t}} = D\bar{\Delta}\bar{A} + \frac{\bar{A}^2}{\bar{I}} - \bar{A} \tag{2.3}$$
$$\frac{\partial \bar{I}'}{\partial \bar{t}} = \bar{\Delta}\bar{I}' + \mu(\bar{A}^2 - \bar{I}) + \sigma \tag{2.4}$$

where $\sigma = \frac{k_I * k_{D_I} * s_I}{s_A^2}$, $\mu = \frac{k_{D_I}}{k_{D_A}}$, $D = \frac{D_A}{D_I}$, $\bar{\Delta} = \partial^2/\partial\bar{x}^2$

In the absence of diffusion, the system has three steady states, which are given by:
$A_0 = 0$, $I_0 = \frac{\sigma}{\mu}$
$A_0 = I_0 = \frac{\mu \pm \sqrt{\mu^2 - 4\mu\sigma}}{2\mu}$
These correspond to two stable points (at $A_0 = 0$ and $A_0 = \frac{\mu + \sqrt{\mu^2 - 4\mu\sigma}}{2\mu}$, with a saddle point at $A_0 = \frac{\mu - \sqrt{\mu^2 - 4\mu\sigma}}{2\mu}$. All the subsequent analyses are performed for the stable steady state with non-zero A_0.
To determine the stability of the steady state, we introduce a small perturbation ($|\delta A_0|, |\delta I_0| << 1$) around steady state:

$$\bar{A} = A_0 + \delta A \tag{2.5}$$
$$\bar{I}' = I_0 + \delta I \tag{2.6}$$

where

$$\delta A = \delta A_0 e^{\omega t} cos(kx) \tag{2.7}$$
$$\delta I = \delta I_0 e^{\omega t} cos(kx) \tag{2.8}$$

k is the wavenumber associated with the spatial perturbation. The system operates in a closed domain ([0,L]) with boundary conditions such that the flux vanishes at the boundaries:

$$\left.\frac{\partial A}{\partial x}\right|_{x=0,x=L} = \left.\frac{\partial I}{\partial x}\right|_{x=0,x=L} = 0 \tag{2.9}$$

Thus, k takes only discrete values: $k_n = n\pi/L$, n = 0,1,2...

Introducing values from (7-10) in (5-6), linearizing the reaction terms and retaining terms up to first order, gives:

$$\begin{pmatrix} \omega + k^2 D - 1 & 1 \\ -2\mu A_0 & \omega + k^2 + \mu \end{pmatrix} \begin{pmatrix} \delta A_0 \\ \delta I_0 \end{pmatrix} = 0 \tag{2.10}$$

The perturbation amplitudes($\delta A_0, \delta I_0$) are different from zero if and only if,

$$\begin{vmatrix} \omega + k^2 D - 1 & 1 \\ -2\mu A_0 & \omega + k^2 + \mu \end{vmatrix} = 0 \tag{2.11}$$

which implies,

$$\omega^2 + \alpha\omega + \beta = 0 \tag{2.12}$$

$$\omega = \frac{-\alpha \pm \sqrt{(\alpha^2 - 4\beta)}}{2}$$

where

$$\alpha = k^2(D+1) + \mu - 1 \tag{2.13}$$
$$\beta = Dk^4 + (\mu D - 1)k^2 + \mu(2A_0 - 1) \tag{2.14}$$

To obtain a diffusion driven instability, it is necessary that the perturbation grows with time i.e. $\mathrm{Re}[\omega(k)] > 0$, and the wavelength of the pattern fits in the spatial domain. Assuming that the the length 'L' of the domain is much larger than the wavelength of the pattern, we can derive conditions for spatial patterning. In this length regime, we consider the wavenumber 'k' as a continuous variable greater than 0. Perturbations grow at this wavenumber if $\mathrm{Re}[\omega(k)] > 0$. This happens if one of the following two conditions is true:
1) $\alpha < 0$
The condition $\alpha < 0$ equates to

$$k^2(D+1) + \mu < 1 \tag{2.15}$$

As k, D and μ are positive constants, $\mu < k^2(D+1) + \mu$. Thus,

$$\mu < k^2(D+1) + \mu < 1 \tag{2.16}$$

If $\mu < 1$, there exist values of k such that inequality (17) is satisfied. This instability can be achieved without invoking diffusion, and thus, is not diffusion driven and does not result in any spatial pattern.

2) $\alpha > 0$ and $\beta < 0$
By the same reasoning as above, requiring $\alpha > 0$ for all k $\in$ R yields,

$$\mu > 1 \tag{2.17}$$

The condition $\beta < 0$ equates to

$$Dk^4 + (\mu D - 1)k^2 + \mu(2A_0 - 1) < 0 \tag{2.18}$$

The left hand side of the above inequality represents a concave parabola in the variable $\gamma = k^2$.

$$f(\gamma) = D\gamma^2 + (\mu D - 1)\gamma + \mu(2A_0 - 1) \tag{2.19}$$

$f(\gamma) < 0 \implies \min(f(\gamma)) < 0$. This minimum of $f(\gamma)$ is given by the value of γ for which

$$\begin{aligned} \frac{df}{d\gamma} &= 0 \\ 2D\gamma + \mu D - 1 &= 0 \\ \gamma &= \frac{1 - \mu D}{2D} \end{aligned} \tag{2.20}$$

As $\gamma = k^2$ and $k^2 > 0$, from equation (22), we get

$$\mu D < 1 \tag{2.21}$$

Now, $\min(f(\gamma)) < 0$ gives

$$D(\frac{1-\mu D}{2D})^2 + (\mu D - 1)(\frac{1-\mu D}{2D}) + \mu(2A_0 - 1) < 0 \tag{2.22}$$

Simplifying equation (24) gives

$$\mu < \frac{(\sqrt{2A_0} - \sqrt{2A_0 - 1})^2}{D} \tag{2.23}$$

From (19) and (25),

$$1 < \mu < \frac{(\sqrt{2A_0} - \sqrt{2A_0 - 1})^2}{D} \tag{2.24}$$

This instability occurs only in the presence of diffusion, and is therefore diffusion driven. Within some finite range of wavenumbers k (k > 0), this instability will form spatial pattern provided the length of reaction domain is large enough to fit the spatial perturbations.

The homogeneous steady state would be stable if $\alpha > 0$ and $\beta > 0$, which, from above analyses equates to:

$$\mu > \frac{(\sqrt{2A_0} - \sqrt{2A_0 - 1})^2}{D} \tag{2.25}$$

To summarize,
1) A diffusion driven instability occurs if $\mu > 1$ & $D < \frac{(\sqrt{2A_0} - \sqrt{2A_0 - 1})^2}{\mu}$
2) System reaches a homogeneous steady state if $\mu > 1$ & $D > \frac{(\sqrt{2A_0} - \sqrt{2A_0 - 1})^2}{\mu}$
(It must be noted that A_0 is a function of μ and σ.)

3 Simulation components

3.1 Simulation domain

All simulations were performed on a 2d lattice of size 190 by 190 pixels. To incorporate a circular colony, a circle of radius 25 pixels was defined at the center of the lattice. Experimentally, the circle is analogous to a circular colony of radius 400μm, and the lattice region outside the circle is analogous to media. $\therefore$ 1 pixel = 16μm.

3.2 Model parameters

The time step for running the simulation(dt) was 0.1. Assuming the unit of time is minutes, dt = 0.1min and the unit of concentration is picomoles(pM). With 1 pixel = 16μm and 1 time step = 0.1min, the parameter values that recapitulate inwards movement of paracrine signaling activities are as follows:

These parameter values are in the same order of magnitude as observed experimentally [3], [1].

Parameters	Values in simulation	Values with units	Parameter meaning
D_A	0.02	$0.85\mu m^2/s$	Diffusion coefficient of activator
D_I	0.4	$17\mu m^2/s$	Diffusion coefficient of inhibitor
k_{d_A}	0.001	$1.6 * 10^{-4}/s$	Degradation rate of activator
k_{d_I}	0.008	$12.8 * 10^{-4}/s$	Degradation rate of inhibitor
s_A	0.01	$1.6 * 10^{-3}/s$	Auto-activation rate of activator
s_I	0.01	$1.6 * 10^{-3}/(s)$(pM)	Rate at which activator activates 1pM inhibitor
k_I	1	1pM	Minimum effective inhibitor concentration

3.3 Turing patterns

Keeping all the parameters unchanged, transition into Turing pattern formation regime is possible by reducing the diffusion constant of the activator (D_A) (equation 26). For the above mentioned parameter set, the critical value of D_A, at which the system bifurcates is $0.0087 pixel^2/(0.1 min) = 0.3712\mu m^2/s$. It must be noted that near the bifurcation point, even within the Turing regime, the activator concentration in the lattice appears to be homogeneous as the difference between values across the lattice is negligible.

References

[1] Uri Alon. *An introduction to systems biology: design principles of biological circuits.* Chapman and Hall/CRC, 2006.

[2] AJ Koch and Hans Meinhardt. Biological pattern formation: from basic mechanisms to complex structures. *Reviews of modern physics*, 66(4):1481, 1994.

[3] Patrick Müller, Katherine W Rogers, Ben M Jordan, Joon S Lee, Drew Robson, Sharad Ramanathan, and Alexander F Schier. Differential diffusivity of nodal and lefty underlies a reaction-diffusion patterning system. *Science*, 336(6082):721–724, 2012.
