# Supplementary material for: Dissecting the dynamics of signaling events in the BMP, WNT, and NODAL cascade during self-organized fate patterning in human gastruloids
Source: PLoS Biol. 2019 Oct 15;17(10):e3000498. doi: 10.1371/journal.pbio.3000498 (PMC6814242; doi:10.1371/journal.pbio.3000498)
Supplement: S2 Model — (PDF) [file pbio.3000498.s030.pdf]

# WNT-NODAL reaction diffusion model

September 18, 2019

Consider a reduced reaction-diffusion model which assumes that the relevant inhibitors remain relatively constant. We obtain a reduced system which only allows the concentration of the two activators WNT and NODAL to vary along with the concentration levels of BMP4. We denote the concentrations of these as $u$, $v$ and $b$ respectively. Inside the stem-cell colony, with circular domain $\{|(x,y)| < r_{\mathrm{rad}}\}$ of radius $r_{\mathrm{rad}}$, we propose the following PDE-model

$$\begin{aligned}\frac{\partial u}{\partial t} &= D_u \Delta u + r_f \left( f(u) - k_u u + k_b b_{\mathrm{data}} + k_p \right) \\ \frac{\partial v}{\partial t} &= D_v \Delta v + r_g \left( g(u,v) - k_v v \right)\end{aligned}$$

$$f(u) = \frac{s_u u^2}{1+\kappa_u u^4}, \qquad g(u,v) = \frac{s_v v}{1+\kappa_v v^2}\left[u + \chi(v)(v-u)\right] \tag{0.1}$$

where $\chi$ is a smooth, monotonic step function, which satisfies $\chi \equiv 0$ for all $v \leq v_{\mathrm{th}} - \delta$ and $\chi \equiv 1$ for all $v \geq v_{\mathrm{th}} + \delta$. Here $v_{\mathrm{th}} > 0$ is a threshold concentration above which NODAL production switches from being WNT dependent to being auto-catalytic. An explicit formula for $\chi$ which approximately satisfies these conditions is

$$\chi(v) = \left[\tanh\left(\frac{v - v_{\mathrm{th}}}{\delta}\right) + 1\right]/2, \qquad 0 < \delta \ll 1 \tag{0.2}$$

Furthermore, the effect of NODAL inhibitors are included via a simplifying saturation term $(1 + \kappa_v v^2)$ in the denominator, that is the growth rate of $v$ is reduced as the concentrations grows large. Also, $b_{\mathrm{data}} = b_{\mathrm{data}}(x,y,t)$ denotes scaled BMP concentration data, averaged over multiple experiments, with $WNT$-growth rate constant $k_b$. The term $k_p$ represents a constant source term which incorporates homogeneous production of WNT in the background.

The nonlinearity $f(u)$ incorporates the autocatalytic production of WNT via the term $s_u u^2$, while including simplified inhibitor dynamics via a saturation term, $(1 + \kappa_u u^4)$, in the denominator, which slow production of WNT as its concentration becomes large.

Also, $r_f$ and $r_g$ are additional scaling constants which compare the relative diffusive length scales of the two concentrations. Finally note that the reaction constants $s_u$ and $s_v$ contain the scaling of the constant inhibitor concentrations.

Furthermore note that outside the cell-colony, $\{|(x,y)| > r_{\mathrm{rad}}\}$ the nonlinear reaction terms are set to zero and $u$ and $v$ degrade with increased rates, $k_u$ and $k_v$. Also, note that for some simulations, we also can model the BMP4 concentration as a diffusive species, satisfying $b \equiv 1$ for some initial time range $(0, t_{\mathrm{loc}})$ (see Heemskerk et. al. 2019) and the equation

$$\begin{cases} b_t = D_b \Delta b, & \text{for } |(x,y)| < r_{\mathbf{loc}} \\ b(x,y,t) \equiv 1, & \text{for } |(x,y)| > r_{\mathbf{loc}} \end{cases} \tag{0.3}$$

where $r_{\mathrm{loc}} \sim r_{\mathrm{rad}} - 50/160$.

We then assign intensities of the various cell-fates, BRA, CDX2, and SOX2, which we denote as $\mathcal{B}, \mathcal{C}, \mathcal{S}$, in the colony as:

$$\mathcal{B} = \chi_{cut}(u)\left(w_{\mathcal{B},u} + w_{\mathcal{B},v}v\right), \qquad \mathcal{C} = b\left(w_{\mathcal{C},b} + w_{\mathcal{C},u}u\right)\left(1 - w_{\mathcal{C},v}v\right), \qquad \mathcal{S} = \left(1 - \chi_{cut}(u)\right)\left(1 - w_{\mathcal{S},b}b\right)$$

where $\chi_{cut}$ is a cut-off or threshold function which is smooth, monotonic, close to zero for $u < 0.7$ and close to one for $u \sim 1$. For definiteness, we take $\chi_{cut}(u) = \frac{\tanh((u-0.95)*15)+1}{2}$. These were formulated from inspecting the summarizing cell-fates experiments under Wnt inhibition and Nodal knockout in Figure 1. For example, let us take the CDX2 density. First, Figure 3(f) shows that BMP inhibition negatively effects CDX2 intensity, so that $\mathcal{C}$ must vary positively with increase in $b$. Next, we see that Wnt-inhibition greatly reduces the production of $\mathcal{C}$, so that $\mathcal{C}$ varies positively with increase in $u$. And then finally, Nodal knockout greatly increases the intensity of CDX2 and so we make $\mathcal{C}$ vary inversely with $v$.

The constants $w_{i,j}$, for $i = \mathcal{C}, \mathcal{B}, \mathcal{S}, j = u, v, b$, were determined to fit numerical data to experimental data, *only* in the circular colony.

Here we assume the following approximate ranges for the various parameters:

| Parameters | Values in simulation | Values with units | Parameter meaning |
|---|---|---|---|
| $D_u$ | $1.5 * 10^{-4}$ | $6.4 * 10^{-3} \mu m^2/s$ | diffusion constant for WNT. |
| $D_v$ | $10^{-3}$ | $4.3 * 10^{-2} \mu m^2/s$ | diffusion constant for NODAL |
| $D_b$ | $10^{-4}$ | $4.3 * 10^{-3} \mu m^2/s$ | diffusion constant for BMP4, |
| $s_u$ | 0.125 | $2.1 * 10^{-3}/(pMs)$ | rate constant for auto-catalytic $u$ equation |
| $s_v$ | 0.225 | $3.8 * 10^{-3}/(pMs)$ | rate constant for $v$ reaction |
| $k_u$ | 0.1 | $1.6 * 10^{-3}/s$ | degradation rate of WNT $u$ in colony |
| $k_v$ | 0.05 | $6.6 * 10^{-4}/s$ | degradation rate of NODAL $v$ in colony |
| $k_b$ | $2 * 10^{-3}$ | $2 * 10^{-4}/s$ | production rate of WNT $u$ from BMP $b$ |
| $k_p$ | $2 * 10^{-2}$ | $3 * 10^{-4} pM/s$ | homogeneous production of $u$ |
| $\kappa_u$ | 0.6 | $0.6/\text{pM}^4$ | saturation constant of WNT, $u$ |
| $\kappa_v$ | 4 | $4/\text{pM}^2$ | saturation constant of NODAL, $v$ |
| $r_f$ | 0.21 | | dimensionless time-scaling constant for WNT $u$ |
| $r_g$ | 1 | | dimensionless time-scaling constant for NODAL $v$ |
| $v_{\text{th}}$ | 0.7 | 0.7 pM | threshold constant for conversion of NODAL production |
| $(w_{\mathcal{C},u}, w_{\mathcal{C},v}, w_{\mathcal{C},b})$ | $(0.8, 0.65, 1)$ | $(0.8, 0.65, 1)/(\text{pM})$ | weights which give the normalized peak intensity for CDX2 |
| $(w_{\mathcal{B},u}, w_{\mathcal{B},v}, w_{\mathcal{B},b})$ | $(1, 0.2, 0)$ | $(1, 0.2, 0)/(\text{pM})$ | weights which give the normalized peak intensity for BMP |
| $(w_{\mathcal{S},b})$ | 0.5 | $0.5/(\text{pM})$ | weights which give the normalized peak intensity for SOX2 |
