# Supplementary material for: Dissecting the dynamics of signaling events in the BMP, WNT, and NODAL cascade during self-organized fate patterning in human gastruloids
Source: PLoS Biol. 2019 Oct 15;17(10):e3000498. doi: 10.1371/journal.pbio.3000498 (PMC6814242; doi:10.1371/journal.pbio.3000498)
Supplement: S1 Text — (PDF) [file pbio.3000498.s041.pdf]

Data:

All the quantification represented in the manuscript requires one of the four categories of processed data outlined below. The data is saved in a cell/structure array in a matlab file format (.mat).

1) Radial intensity profiles:

a) Fig 1A-C, Fig 2A, B, H, Fig 4C, Fig 5J, Fig 6-9
Quantification represents radial intensity profiles for each colony (computed as outlined in S9 Fig) for each of the conditions indicated in the specific figure. The data is saved in an m by n (m*n) array, where each row (m) represents the colony and each column (n) represents the midpoint of the bin (bin = range of distance from the edge of the colony) used to compute the average intensity. The numeric values (in um) for the bins are provided separately in the output files.

b) Fig 3D, Fig 5B,F
For time-lapse movies of WNT signaling dynamics, β-catenin radial profile of individual colonies is saved in a 3D array, where the first two dimensions are same as before and the third dimension corresponds to imaging time point. Mean radial profile consisting the colony wide average and the threshold intensity for computing signaling movement is saved separately.

Data plotted in Figures 3E, 5C, D, H, I can be easily derived from the saved data, and is thus, not provided separately.

2) RNA sequencing analyses:

Differential expression analyses results in Fig 2C are saved in a cell array with each cell containing information on genes differentially expressed in the samples compared. The information consists of gene names, average FPKM read counts for the samples compared, and the absolute fold change. Quantification in Fig 2D-F requires additional information saved in S1_Table.

3) Cell movement statistics:

Cell tracking data quantified in Fig 4B,D is saved in a cell array called cellMovementStats. Each cell in the array represents information about one cell in the movie in the form of an m by n array. Here, m represents the imaging time point and n represents the cell's features. The order of features is as: [xPosition yPosition colonyId lineageId trackId tStart tEnd DistanceFromCenter colonyPartId(1/2)].

<u>xPosition:</u> X coordinate of cell's centroid
<u>yPosition:</u> Y coordinate of cell's centroid
<u>colonyId:</u> colony Id corresponding to the cell

lineageId: A cell and its descendants have one lineage Id.
trackId: Each individual cell has a unique trackId
tStart: First time point when the cell appears
tEnd: Last time point when the cell appears
Distance from Center: Distance (in microns) of the cells from the colony center.
colonyPartId(1/2): Identifier for the colony part used for tracking. To facilitate tracking, each colony was divided into two parts such that there is no crossover of labeled cells across parts, and then each part was separately used for cell tracking.

4) Simulations:

For Fig 10A-D, simulated values are saved in a 1 by n array, where each column represents distance from the colony edge. The distances are saved separately in the variable xValues.
